# Inhibitors of dihydroorotate dehydrogenase cooperate with molnupiravir and N4-hydroxycytidine to suppress SARS-CoV-2 replication

**DOI:** 10.1016/j.isci.2022.104293

**Published:** 2022-04-25

**Authors:** Kim M. Stegmann, Antje Dickmanns, Natalie Heinen, Claudia Blaurock, Tim Karrasch, Angele Breithaupt, Robert Klopfleisch, Nadja Uhlig, Valentina Eberlein, Leila Issmail, Simon T. Herrmann, Amelie Schreieck, Evelyn Peelen, Hella Kohlhof, Balal Sadeghi, Alexander Riek, John R. Speakman, Uwe Groß, Dirk Görlich, Daniel Vitt, Thorsten Müller, Thomas Grunwald, Stephanie Pfaender, Anne Balkema-Buschmann, Matthias Dobbelstein

**Affiliations:** 1 Institute of Molecular Oncology, Göttingen Center of Molecular Biosciences (GZMB), University Medical Center Göttingen, Justus von Liebig Weg 11, 37077 Göttingen, Germany; 2 Department of Molecular and Medical Virology, Ruhr University Bochum, Bochum, Germany; 3 Friedrich-Loeffler-Institut, Federal Research Institute for Animal Health, Greifswald-Insel Riems, Germany; 4 Institute of Veterinary Pathology, Freie Universität Berlin, Berlin, Germany; 5 Fraunhofer Institute for Cell Therapy and Immunology IZI, Leipzig, Germany; 6 Department of Molecular Biochemistry, Ruhr University Bochum, Bochum, Germany; 7 Immunic AG, Gräfelfing, Germany; 8 Friedrich-Loeffler-Institut, Institute of Novel and Emerging Infectious Diseases, Greifswald-Insel Riems, Germany; 9 Friedrich-Loeffler-Institut, Institute of Animal Welfare and Animal Husbandry, Celle, Germany; 10 Institute of Biological and Environmental Sciences, University of Aberdeen, Aberdeen, UK; 11 Institute of Medical Microbiology and Virology, Göttingen Center of Molecular Biosciences (GZMB), University Medical Center Göttingen, Göttingen, Germany; 12 Max Planck Institute for Biophysical Chemistry, Göttingen, Germany; 13 Institute of Psychiatric Phenomics and Genomics (IPPG), Organoid Laboratory, University Hospital, LMU Munich, Munich, Germany

**Keywords:** Virology, Drugs

## Abstract

The nucleoside analog N4-hydroxycytidine (NHC) is the active metabolite of the prodrug molnupiravir, which has been approved for the treatment of COVID-19. SARS-CoV-2 incorporates NHC into its RNA, resulting in defective virus genomes. Likewise, inhibitors of dihydroorotate dehydrogenase (DHODH) reduce virus yield upon infection, by suppressing the cellular synthesis of pyrimidines. Here, we show that NHC and DHODH inhibitors strongly synergize in the inhibition of SARS-CoV-2 replication *in vitro*. We propose that the lack of available pyrimidine nucleotides upon DHODH inhibition increases the incorporation of NHC into nascent viral RNA. This concept is supported by the rescue of virus replication upon addition of pyrimidine nucleosides to the media. DHODH inhibitors increased the antiviral efficiency of molnupiravir not only in organoids of human lung, but also in Syrian Gold hamsters and in K18-hACE2 mice. Combining molnupiravir with DHODH inhibitors may thus improve available therapy options for COVID-19.

## Highlights

• Molnupiravir and DHODH inhibitors are approved drugs, facilitating clinical testing
• The combination may allow lower drug doses to decrease possible toxic effects
• Inhibitors of nucleotide biosynthesis may boost antiviral nucleoside analogs

## Graphical abstract

**Figure:**
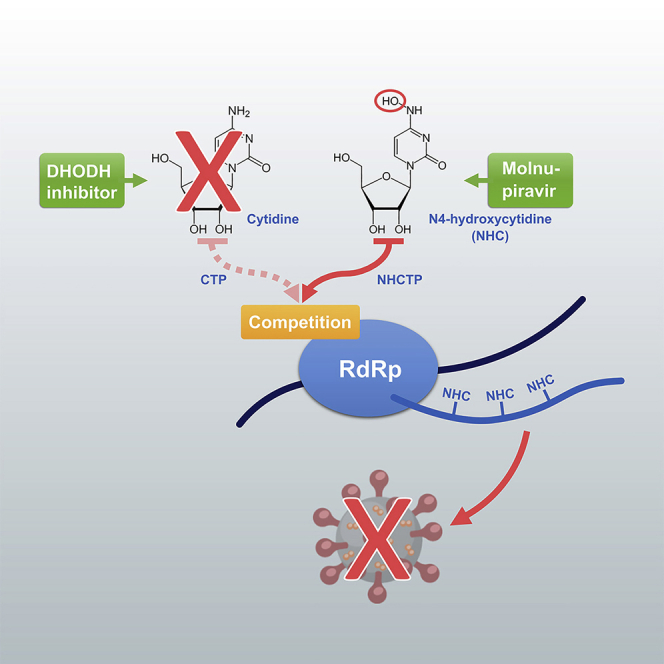

## Introduction

During the combat of the COVID-19 pandemic, a number of vaccine approaches have been established successfully, while efficient therapeutics are still urgently needed (Doherty, 2021). Clinically evaluated therapies include the use of steroids (Horby et al., 2021; Tomazini et al., 2020), the protease inhibitor nirmatrelvir that is contained in Paxlovid (Wang and Yang, 2021), and the nucleoside analog remdesivir (Beigel et al., 2020; Goldman et al., 2020). Upon triphosphorylation at their 5′ positions, antiviral nucleoside analogues antagonize virus propagation, by interfering with the activity of the viral RNA-dependent RNA polymerase and/or by compromising the function of the newly synthesized viral genomes through mutagenesis (Pruijssers and Denison, 2019).

Besides remdesivir, other nucleoside analogs showed promising antiviral effects against SARS-CoV-2. Most notably, molnupiravir, also known as EIDD-2801 or MK-4482, is the prodrug of N4-hydroxycytidine (NHC), or EIDD-1931 (Cox et al., 2021; Painter et al., 2021; Sheahan et al., 2020; Wahl et al., 2020, 2021). In comparison to cytidine, NHC has the same structure but carries a hydroxylated amino group (nitrogen 4) at the pyrimidine base. This does not impair the incorporation of triphosphorylated NHC into nascent RNA by the viral RNA-dependent RNA polymerase (Kabinger et al., 2021). However, owing to a tautomeric interconversion within the NHC base, the incorporation of NHC into virus RNA leads to erroneous RNA replication (Jena, 2020). NHC can base pair with guanosine, but also with adenosine, thus leading to multiple errors in the subsequently synthesized viral RNA genomes and resulting in replication-deficient virus particles. Molnupiravir is active against SARS-CoV-2 replication *in vitro* and *in vivo* (Rosenke et al., 2021; Sheahan et al., 2020; Wahl et al., 2021), and this includes the recently emerged Omicron variant (Prince et al., 2021; Vangeel et al., 2021). It also prevents SARS-CoV-2 transmission in the ferret model (Cox et al., 2021), and it was found clinically effective in large-scale clinical trials (Jayk Bernal et al., 2021; Painter et al., 2021), exemplified by the trials registered at clinicaltrials.gov with the numbers NCT04575584, NCT04575597 and NCT04405739, leading to approval in the UK. However, although the drug reduced the risk of hospitalization of patients with COVID-19 roughly by 30% (Jayk Bernal et al., 2021), this still leaves ample space for further improvement.

Besides immunosuppression and direct interference with virus replication, an alternative approach of treatment against SARS-CoV-2 aims at reducing the cellular synthesis of nucleotides, thereby indirectly impairing the synthesis of viral RNA. We (Stegmann et al., 2021a) and others (Caruso et al., 2021; Zhang et al., 2021) have previously reported the high demand on cellular nucleotide biosynthesis during SARS-CoV-2 infection, resulting in an antiviral effect of folate antagonists, which impair purine synthesis. Moreover, in the context of nucleotide biosynthesis, the inhibition of dihydroorotate dehydrogenase (DHODH) represents an attractive strategy to antagonize SARS-CoV-2 replication. DHODH catalyzes a key step during pyrimidine synthesis. Unlike all cytosolic enzymes involved in this metabolic pathway, DHODH localizes to the inner mitochondrial membrane, where it transfers reduction equivalents from dihydroorotate to ubiquinone moieties of the respiration chain. As a result, orotate becomes available for the subsequent synthesis steps to obtain uridine monophosphate and later cytidine triphosphate. A number of DHODH inhibitors have become available for clinical testing or were even approved for therapy (Munier-Lehmann et al., 2013), mostly to treat autoimmune diseases, due to their selective inhibition of hyperactive immune cells. Recently, however, some DHODH inhibitors were successfully tested with regard to their efficacy in preventing the replication of viruses (Hoffmann et al., 2011; Zhang et al., 2012), including SARS-CoV-2 (Calistri et al., 2021; Hahn et al., 2020; Luban et al., 2021; Xiong et al., 2020). One DHODH inhibitor, IMU-838 (Vidofludimus calcium), was further clinically evaluated for COVID-19 therapy in hospitalized patients and was found effective according to secondary criteria, e.g. time to clinical improvement or viral burden (CALVID-1, trial identifier NCT04379271; NCT04516915).

We hypothesized that the suppression of pyrimidine synthesis should increase the ratio of NHC triphosphate versus cytidine triphosphate in infected cells, thus enhancing the incorporation of NHC into the viral RNA and resulting in the production of replication-deficient viral particles. Preliminary results that we (Stegmann et al., 2021b) and others (Schultz et al., 2022) presented on a pre-publication server revealed first *in vitro*-evidence of drug synergism between NHC and DHODH inhibitors, supporting our hypothesis and encouraging its further evaluation. We now found that the combination of NHC and DHODH inhibitors resulted in profoundly synergistic suppression of SARS-CoV-2 replication *in vitro*; DHODH inhibition also improved the performance of molnupiravir in two animal model systems, thus presenting a potential treatment strategy.

## Results

### NHC and DHODH inhibitors cooperate to interfere with SARS-CoV-2 replication in cultured cells, without signs of cytotoxicity

**Figure 1:**
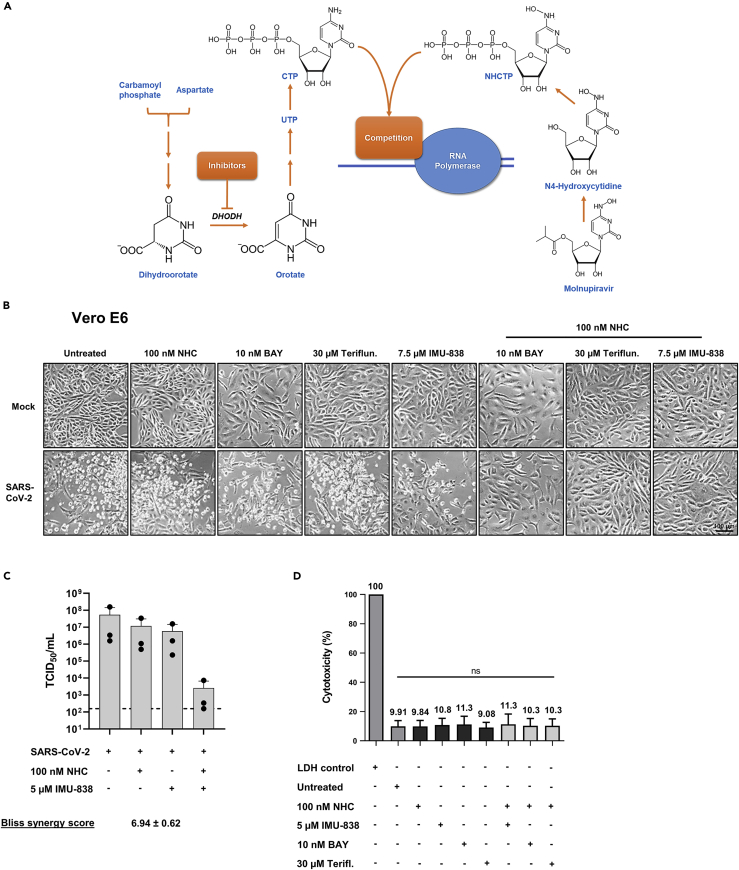
The combination of N4-hydroxycytidine (NHC) and inhibitors of dihydroorotate dehydrogenase (DHODH) strongly impairs SARS-CoV-2 replication without detectable cytotoxicity (A) Mechanistic concept for the synergistic inhibitory effect of DHODH inhibitors and N4-hydroxycytidine (NHC) on SARS-CoV-2 RNA replication. Biosynthesis of pyrimidines starts with carbamoyl phosphate and aspartate to form dihydroorotate. Dihydroorotate is further oxidized to orotate by dihydroorotate dehydrogenase (DHODH) and later converted to uridine triphosphate (UTP) and cytidine triphosphate (CTP). Molnupiravir is the prodrug of NHC, which is further converted to the corresponding triphosphate (NHCTP), which competes with CTP for incorporation into nascent virus RNA. The suppression of CTP synthesis by inhibitors of DHODH is expected to enhance the incorporation of NHCTP into the viral RNA, causing false incorporation of nucleotides in subsequent rounds of replication. (B) Reduced cytopathic effect (CPE) by NHC and DHODH inhibitors. Vero E6 cells were treated with drugs or the DMSO control for 24 h, inoculated with SARS-CoV-2 (strain GOE_001), and further incubated in the presence of the same drugs for 48 h. Cell morphology was assessed by phase contrast microscopy. Note that the CPE was readily visible in virus-infected cells, in DMSO-treated cells and also when cells had been treated with either drug alone. However, the CPE was observed only to a far lesser extent when the cells had been incubated with both NHC and DHODH inhibitors. Bar, 100 μm. (C) Reduction of the median tissue culture infectious dose (TCID_50_) by the combination of NHC and the DHODH inhibitor IMU-838. Vero E6 cells were treated with NHC, IMU-838, or the combination of both compounds for 24 h before infection, and then throughout the time of infection. Cells were infected with SARS-CoV-2, strain hCoV-19/Germany/BY-Bochum-1/2020 (MOI 0.1), and further incubated for 48 h. The supernatant was titrated to determine the TCID_50_/mL (mean with SD, n = 3; logarithmic scale). (D) Lack of measurable cytotoxicity by NHC and DHODH inhibitors. Vero E6 cells were treated with NHC and/or IMU-838, BAY2402234, and teriflunomide at the indicated concentrations for 72 h. The release of lactate dehydrogenase (LDH) to the supernatant was quantified by bioluminescence as a readout for cytotoxicity. The percentages reflect the proportion of LDH released to the media, compared to the overall amount of LDH in the cells (LDH control) (mean with SD, n = 3).

The biosynthesis of pyrimidines is crucial for RNA replication (Figure 1A). The enzyme dihydroorotate dehydrogenase (DHODH) catalyzes the oxidation of dihydroorotate to orotate, which is a precursor of cytidine triphosphate (CTP). In the presence of N4-hydroxycytidine (NHC), its active metabolite NHCTP competes with CTP for incorporation into nascent RNA (Gordon et al., 2021). We hypothesized that the suppression of cellular CTP synthesis by DHODH inhibitors will favor the incorporation of NHCTP into newly synthesized SARS-CoV-2 RNA, and will thus potentiate the antiviral efficacy of NHC. To test this, we combined both drugs for treatment of Vero E6 cells prior to infection with SARS-CoV-2. We applied NHC and the DHODH inhibitors BAY2402234, teriflunomide and IMU-838, at concentrations that only moderately suppressed virus replication as single treatments. Accordingly, neither NHC nor DHODH inhibitors alone grossly affected the development of a cytopathic effect (CPE) caused by SARS-CoV-2. Strikingly, however, the combination of NHC and DHODH inhibitors was far more efficient in preventing CPE (Figure 1B), and it reduced virus yield more than 1000-fold, as determined by the median tissue culture infectious dose (TCID_50_/mL) of the supernatant (Figure 1C). Combining the drugs did not produce morphologic signs of cytotoxicity in non-infected cells (Figure 1B) and did not grossly augment the release of lactate dehydrogenase (LDH) into the culture supernatant (Figures 1D, S1A, and S1B) or reduce cell viability (Figure S1C). Hence, the drug combination interferes with CPE and virus yield, without displaying any detectable cytotoxic effects.

### Combinations of NHC and DHODH inhibitors synergistically reduce viral RNA yield upon infection with SARS-CoV-2

**Figure 2:**
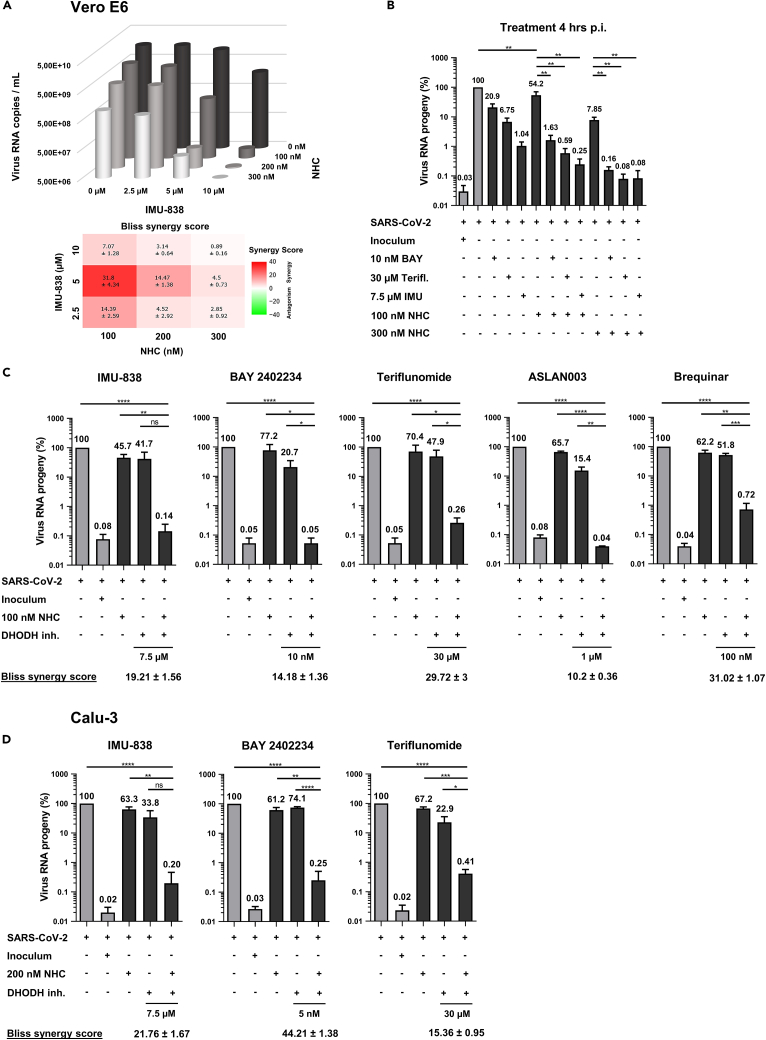
Strong synergism of NHC and DHODH inhibitors to diminish the release of SARS-CoV-2 RNA from cultured cells (A) Reduced release of viral RNA upon combined treatment with NHC and IMU-838. Vero E6 cells were treated with NHC and/or IMU-838, and infected as in Figure 1. A sample of the inoculum was preserved for RNA preparation. At 48 h post infection (p.i.), RNA was isolated from the cell supernatants, followed by quantitative RT-PCR to detect viral RNA and determine the amount of SARS-CoV-2 RNA copies per mL (mean, n = 3). The synergy score was calculated using the Bliss independence model. Data are presented as mean ± SEM. A Bliss score >10 is generally considered to reveal strong drug synergism. (B) Diminished virus RNA progeny by NHC and DHODH inhibitors even when added 4 h after SARS-CoV-2 infection. Vero E6 cells were infected as described in Figure 1 and treated with NHC and/or DHODH inhibitors at 4 h post infection (p.i.). RNA was isolated from the cell supernatants, and SARS-CoV-2 RNA was quantified by qRT-PCR. The amount of RNA found upon infection without drug treatment was defined as 100%, and the other RNA quantities were normalized accordingly. RNA was also isolated from the virus inoculum used to infect the cells. Note that the combination treatment reduced SARS-CoV-2 replication to a greater extent compared to single drug treatments even when applied 4 h p.i. (mean with SD, n = 3). For p values, see Figure S2C. (C) Reduced virus RNA progeny in the presence of NHC and various DHODH inhibitors. Vero E6 cells were treated with drugs and/or infected as in Figure 1, followed by quantitative detection of SARS-CoV-2 RNA. The drug combinations were found capable of reducing virus RNA yield by more than 100-fold as compared to single drug treatments (mean with SD, n = 3). (D) In Calu-3 cells, too, the combination of NHC and the DHODH inhibitors IMU-838, BAY2402234, or teriflunomide strongly reduced the amount of viral RNA released to the supernatant (mean with SD, n = 3).

To assess the synergy of NHC and DHODH inhibitors for reducing virus propagation, we combined different concentrations of the DHODH inhibitor IMU-838 and NHC. Upon infection with SARS-CoV-2, we determined their impact on the release of viral RNA and calculated a synergy score using the Bliss independence model (Figure 2A). IMU-838 and NHC were added 24 h before infection (Figure 2A), at the time of infection (Figures S2A and S2B), or 4 h after infecting the cells (Figures 2B and S2C). The Bliss score revealed strong synergy for a subset of the drug combinations (Figure 2A). Moreover, we combined each of the DHODH inhibitors IMU-838, BAY2402234, teriflunomide, ASLAN003, and brequinar with NHC and quantified the amount of viral RNA released into the cell culture supernatant (Figure 2C). Strikingly, the combination treatment diminished SARS-CoV-2 RNA progeny up to 400-fold as compared to single drug treatment, and up to 1000-fold as compared to untreated controls, and the indices reflected profound synergy of the drugs as determined by the Bliss independence model. This effect was not only seen in Vero E6 cells but also in Calu-3 cells (Figure 2D), a human lung cancer cell line used to model bronchial epithelia (Kreft et al., 2015) and susceptible to infection with SARS-CoV-2 (Saccon et al., 2021). Hence, NHC and DHODH inhibitors synergistically antagonize SARS-CoV-2 replication in two different cell lines.

### Distinctly reduced accumulation of viral proteins in SARS-CoV-2-infected cells upon treatment with NHC and DHODH inhibitors

**Figure 3:**
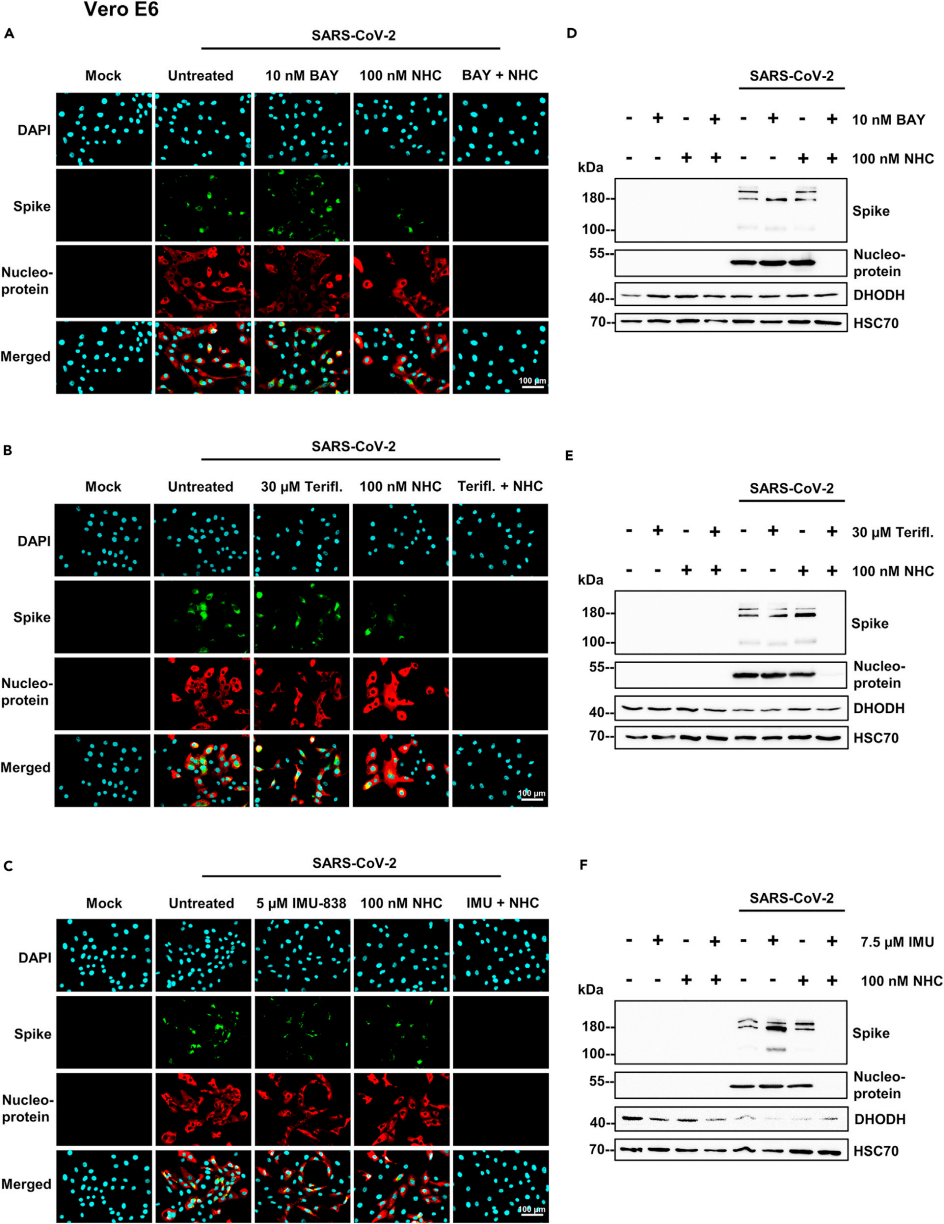
Synergistic reduction of viral protein synthesis by NHC and DHODH inhibitors (A–C) Representative images showing the reduction of viral protein synthesis by NHC and BAY2402234 (A), teriflunomide (B), or IMU-838 (C). Vero E6 cells were treated and infected with SARS-CoV-2 as in Figure 1. Cell nuclei were stained with 4′,6-Diamidino-2-phenylindole (DAPI), and the SARS-CoV-2 spike and nucleoprotein were detected by immunofluorescence. Bar, 100 μm. (D–F) Reduced viral protein synthesis in the presence of NHC and DHODH inhibitors. Upon drug treatment and/or infection of Vero E6 cells as in Figure 1, the viral spike and nucleoprotein as well as DHODH and HSC70 (loading control) were detected by immunoblot analysis. The absence of a signal corresponding to spike or nucleoprotein in non-infected samples ensures the specificity of the antibodies for virus proteins.

Besides infectious units and virus RNA, the synthesis of virus proteins in infected cells is an important readout for virus propagation. Therefore, we detected viral proteins upon treatment of Vero E6 cells with NHC and/or BAY2402234, teriflunomide or IMU-838, and infection with SARS-CoV-2. The frequency at which we detected the viral spike protein and the nucleoprotein in the cells was severely reduced upon the combined treatment, but far less by the single treatments, as determined by immunofluorescence microscopy (Figures 3A–3C). Correspondingly, immunoblot analyses revealed that the levels of both viral proteins were strongly reduced by the drug combination (Figures 3D–3F), whereas the single drugs at the same concentrations were much less efficient. The blots also revealed that DHODH levels were not detectably affected by the drugs. Taken together, the detection of virus proteins further confirmed that the drug combination suppresses virus propagation to a far higher degree than the single drugs in cultured cells.

### The combination of NHC and DHODH inhibitors also reduces the replication of the SARS-CoV-2 variants B.1.1.7/Alpha, B.1.351/Beta, and B.1.617.2/Delta

**Figure 4:**
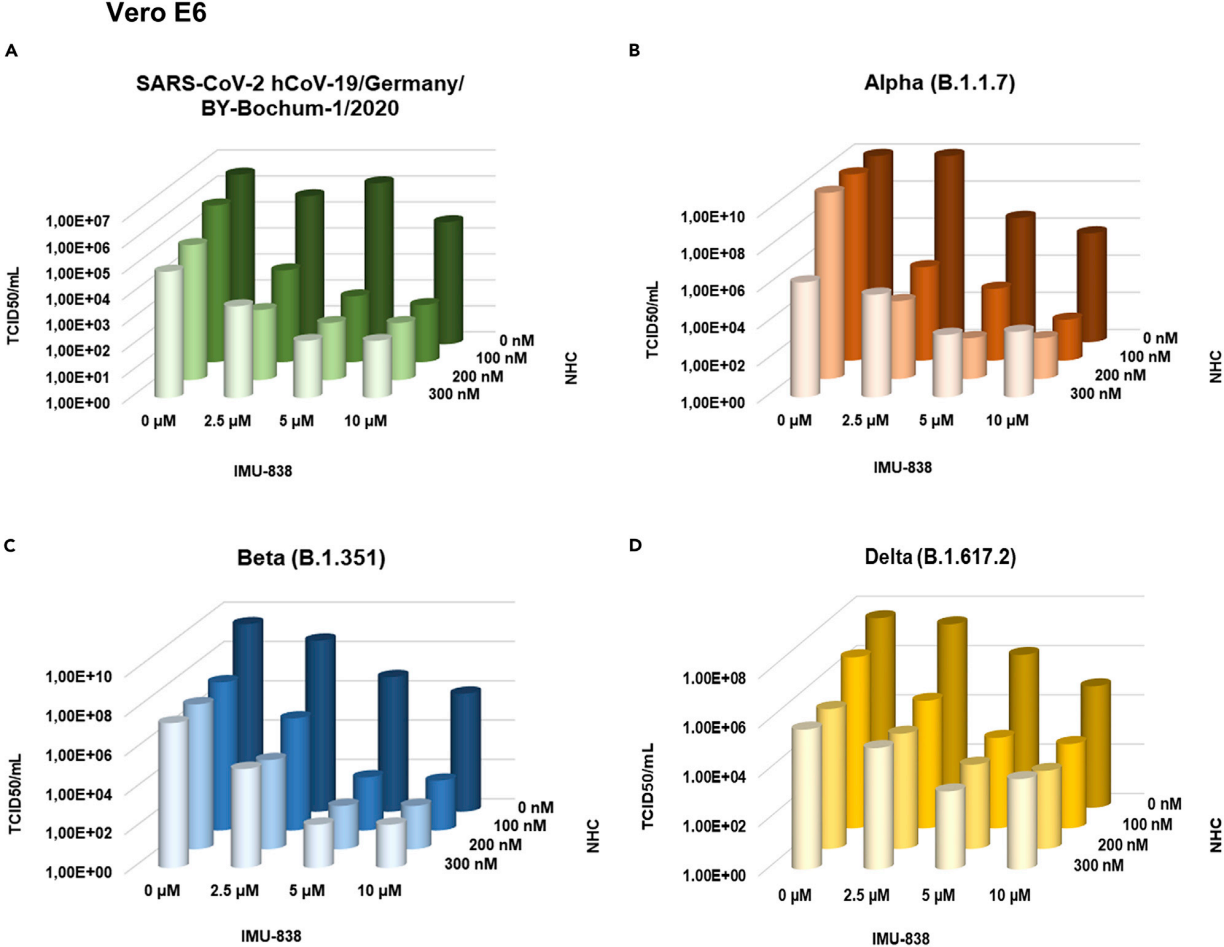
The combination of NHC with DHODH inhibitors synergistically reduces the replication of SARS-CoV-2 variants in Vero E6 cells (A–D) The combination of NHC with IMU-838 synergistically reduced the progeny of SARS-CoV-2 variants. The TCID_50_ of virus progeny was determined upon treatment with NHC and DHODH inhibitors, and infection with the original SARS-CoV-2 or the SARS-CoV-2 variants Alpha, Beta, and Delta. Vero E6 cells were treated with drugs for 24 h before and then throughout the infection. Cells were infected with original SARS-CoV-2 (hCoV-19/Germany/BY-Bochum-1/2020, (A)), Alpha (B.1.1.7, (B)) Beta (B.1.351, (C)), or Delta (B.1.617.2, (D)) (MOI 0.1) and further incubated for 48 h. The supernatant was titrated to determine the TCID_50_/mL. Note that all variants responded similarly to the original strain, indicating that the drug combination is effective against SARS-CoV-2 variants.

As the pandemic proceeded, new variants emerged, with potentially higher infectivity and immune-escape properties (Tegally et al., 2021; Wang et al., 2021a). To ensure the suitability of the proposed drug combination against these variants, we assessed their replication in the presence of NHC and/or DHODH inhibitors. The variants of concern Alpha (B.1.1.7), Beta (B.1.351), and Delta (B.1.617.2) responded similarly as compared to the SARS-CoV-2 wild type (Figures 4A–4D and S3). Thus, the genomic alterations of the variants do not confer any detectable resistance against the drugs or their combination.

### Uridine and cytidine rescue virus replication in the presence of NHC and DHODH inhibitors

**Figure 5:**
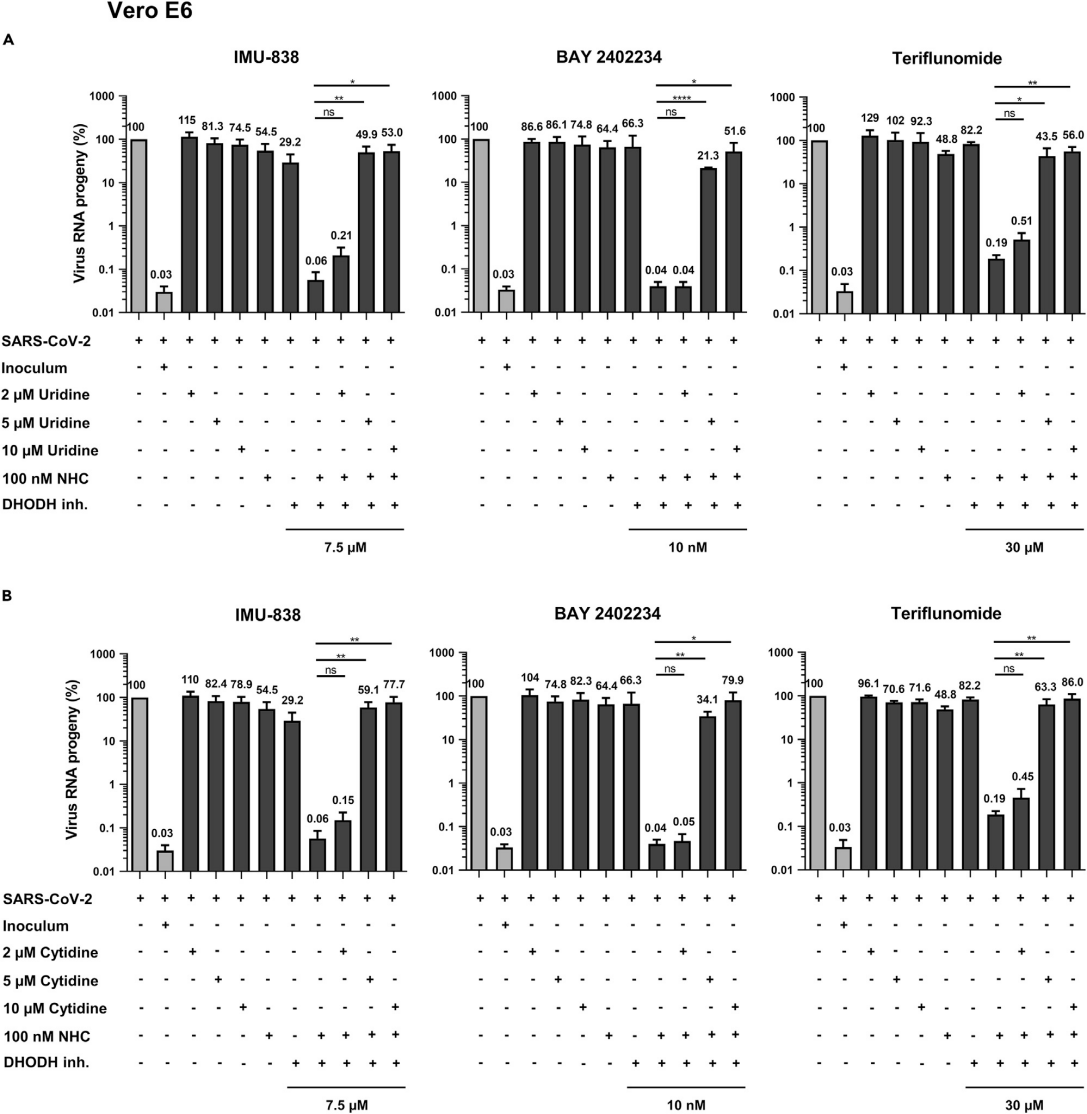
Uridine as well as cytidine rescue SARS-CoV-2 replication in the presence of NHC and DHODH inhibitors (A) The antiviral effect of DHODH inhibitors combined with NHC can be reverted by uridine. Vero E6 cells were treated with drugs and inoculated with SARS-CoV-2 as in Figure 1. On top of the drugs, where indicated, uridine was added to the cell culture media, at concentrations of 2, 5, or 10 μM. SARS-CoV-2 propagation was still diminished by NHC and DHODH inhibitors despite 2 μM uridine levels, but rescued in the presence of 5 or 10 μM uridine (mean with SD, n = 3), in agreement with the mechanism outlined in Figure 1A. For p values, see Figure S4A. (B) Restored SARS-CoV-2 replication by cytidine, in the presence of NHC and DHODH inhibitors. The experiment was carried out as in (A), with the addition of cytidine instead of uridine. 5 or 10 μM cytidine restored virus replication in the presence of the drugs (mean with SD, n = 3), further confirming the mechanism outlined in Figure 1A. For p values, see Figure S4B.

According to our initial considerations (Figure 1A), we suspected that DHODH inhibitors potentiate the efficacy of NHC by suppressing the levels of endogenous pyrimidine nucleotides. We now performed rescue experiments to support this model. To elucidate the mechanism of interference with SARS-CoV-2 replication by the drug combinations, we added pyrimidine nucleosides to the culture media. We supplied uridine (Figures 5A and S4A) or cytidine (Figures 5B and S4B) to Vero E6 cells along with the DHODH inhibitors IMU-838, BAY2402234, or teriflunomide, combined with NHC. The addition of 5 or 10 μM uridine, or the same concentration of cytidine, prevented the inhibition of SARS-CoV-2 replication by the combination treatment. The results are in accordance with the reduced levels of UTP and CTP upon DHODH inhibition that were reported previously (Liu et al., 2020). This strongly suggests that the synergism of NHC with DHODH inhibitors can be explained by competition of NHC with endogenous pyrimidine nucleosides for incorporation into nascent viral RNA, as we had hypothesized initially (Figure 1A).

### DHODH inhibitors and NHC cooperate to reduce SARS-CoV-2 replication in a human lung organoid model

**Figure 6:**
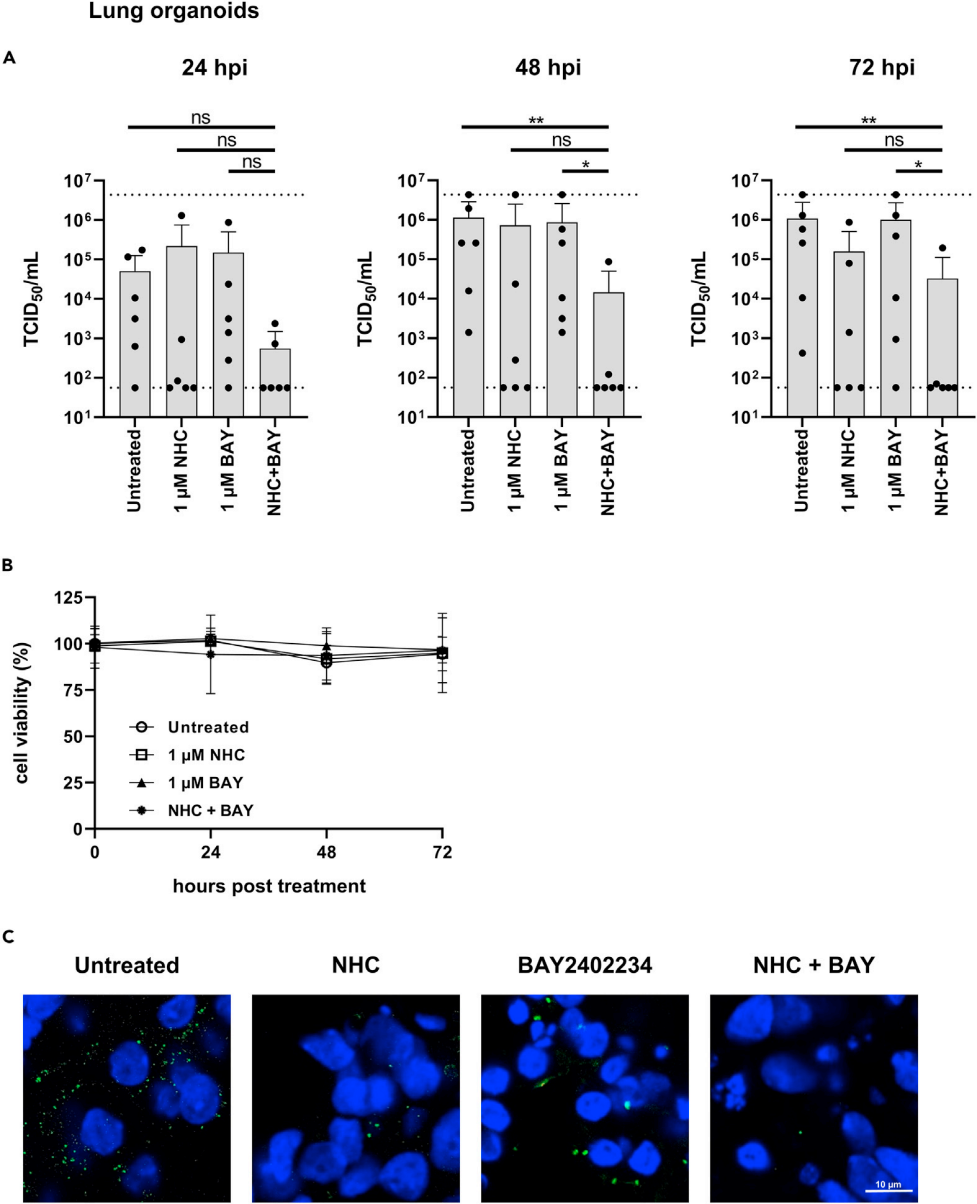
Reduced SARS-CoV-2 propagation and dsRNA formation by NHC and BAY2402234 in human lung organoids (A) Reduced TCID_50_ by NHC and the DHODH inhibitor BAY2402234. Human stem cell-derived lung organoids were sliced and treated with 1 μM NHC and/or 1 μM BAY2402234 for 24 h before and then throughout the time of infection. Organoid slices were infected with 35,000 PFU per well and further incubated for 24, 48, or 72 h. The supernatant was titrated to determine the TCID_50_/mL (mean with SD, n = 6). Statistical evaluation was performed using the Mann-Whitney U test. (B) Cell viability of lung organoids was not detectably affected by NHC and/or the DHODH inhibitor BAY2402234. The release of lactate dehydrogenase (LDH) to the supernatant was quantified by bioluminescence as a readout for cytotoxicity and cell viability as in Figure 1D (mean with SD, n = 6). (C) Representative images showing the reduction of viral double-stranded RNA (dsRNA) formation in lung organoid cells upon treatment with NHC and BAY2402234. Human lung organoid slices were treated and infected as in (A). For immunofluorescence analysis, samples were permeabilized and subjected to staining with an antibody against dsRNA (green dots within cytoplasms). Cell nuclei were stained with Hoechst33342. Bar, 10 μm.

So far, we had performed all experiments in cultured cell lines. The limitation of such experiments consists in their dissimilarity to bronchial and lung epithelia, i.e. the primary sites for SARS-CoV-2 in humans. For a model closer to these primary infection sites, we infected lung organoids derived from human-induced pluripotent stem cells (iPSCs) upon treatment with single and combined drugs. Here again, virus replication was strongly reduced by the combination of both drugs 24, 48, and 72 h.p.i (Figure 6A), with tolerable cytotoxicity (Figure 6B). In parallel, the rate of cells that contained double-stranded RNA derived from the virus was drastically reduced by the drug combination (Figure 6C), further arguing that NHC along with DHODH inhibitors diminishes virus replication in an *in vitro* model of human lung tissue. Notably, however, the effects did not reach the levels of statistically significant synergy, perhaps due to the higher variations in virus yield when using primary organoids rather than cell lines for infection assays.

### The drug combination ameliorates COVID-19 in a hamster model

**Figure 7:**
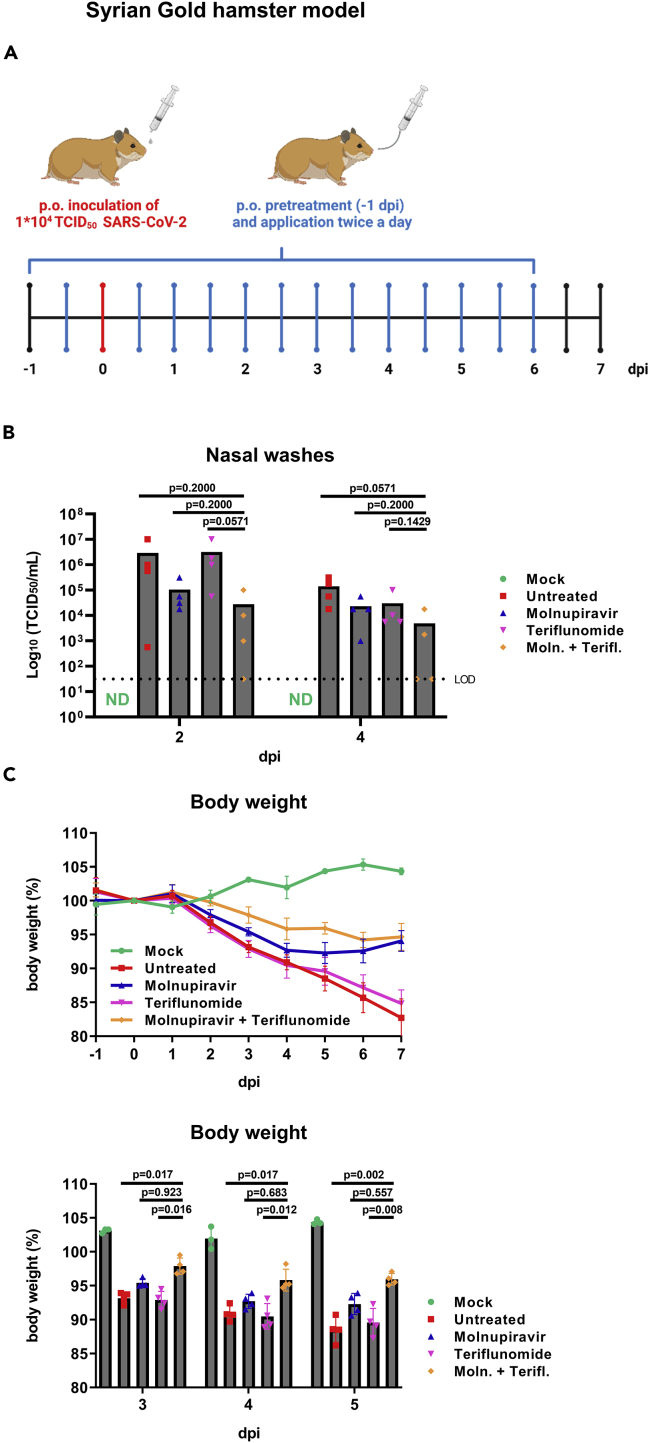
DHODH inhibitors cooperate with Molnupiravir for treating COVID-19 in Syrian Gold hamsters (A) Treatment and infection scheme (drawn with BioRender.com). Male Syrian Gold hamsters (n = 4) were treated with 250 mg/kg molnupiravir alone, 10 mg/kg teriflunomide alone, or a combination of both, administered twice a day, starting 24 h before inoculation until six days post inoculation with 1∗10^4^ TCID_50_ SARS-CoV-2. (B) Virus load within nasal washes, determined by TCID_50_. Nasal washes were obtained at days 2 and 4 post infection (p.i.), and titrated to determine the content of virus. On average, virus load was reduced by 1–2 orders of magnitude as compared to non-treated animals, and the drug combination yielded the strongest reduction. Statistical evaluation was performed using the Mann-Whitney U test (mean with SD, n = 4). (C) Decreased loss in body weight of SARS-CoV-2-infected hamsters in the presence of molnupiravir and teriflunomide. Hamsters were treated and infected as in A, and body weights were documented daily for seven days. Data points represent the body weight of each animal (% of weight at day 0, mean with SD, n = 4). Statistical analysis was performed by one-way ANOVA followed by post hoc Tukey tests (p < 0.05) to reveal that the drug combination was statistically more effective than single drugs on days 3 through 5 post infection. For p values, see Figure S5C.

Syrian Gold hamsters represent a well-established and acknowledged animal model of human SARS-CoV-2 infections. To evaluate the therapeutic effect of the drug combination in this system, we infected Syrian Gold hamsters with SARS-CoV-2 while treating them orally with molnupiravir (the prodrug of NHC) and/or the DHODH inhibitor teriflunomide (Figure 7A). Teriflunomide was used in the *in vivo* models because it is more effective on rodent DHODH compared to e.g. IMU-838, which is more potent on human DHODH (Muehler et al., 2020b). Similarly, the pharmacokinetics (PK) of teriflunomide was favorable in hamsters and mice according to our analyses (Figure S5A), whereas the PK of IMU-838 was previously characterized in humans (Muehler et al., 2020a). Upon drug treatment, the hamsters had reduced virus titers in nasal washes, particularly with the combined therapy (Figure 7B). Infectious virus was no longer detectable in any of the animals at the end of the experiment 7 days p.i (Figure S5B). The weight of the animals typically drops with the progression of the disease (Francis et al., 2021; Imai et al., 2020); using this readout, we observed that the course of COVID-19 was milder when treating the animals with the drugs under study. Of note, the drug combination was superior to the single drugs in the course of infection, as revealed by the weight of the animals at days 3 through 5 post infection (Figures 7C and S5C). Determination of lung pathology (Figures S6A–S6C) as well as daily energy expenditure (Figures S6D) supported these findings. In conclusion, combining molnupiravir and a DHODH inhibitor proved effective in the hamster model of COVID-19. However, the degree of drug synergy was not comparable to the *in vitro* studies using cultured cells. We speculate that the activity of the DHODH inhibitor might be compromised somewhat by the uridine present in the serum of animals, in agreement with our results shown in Figure 5.

### NHC and a DHODH inhibitor suppress SARS-CoV-2 infection of transgenic mice

**Figure 8:**
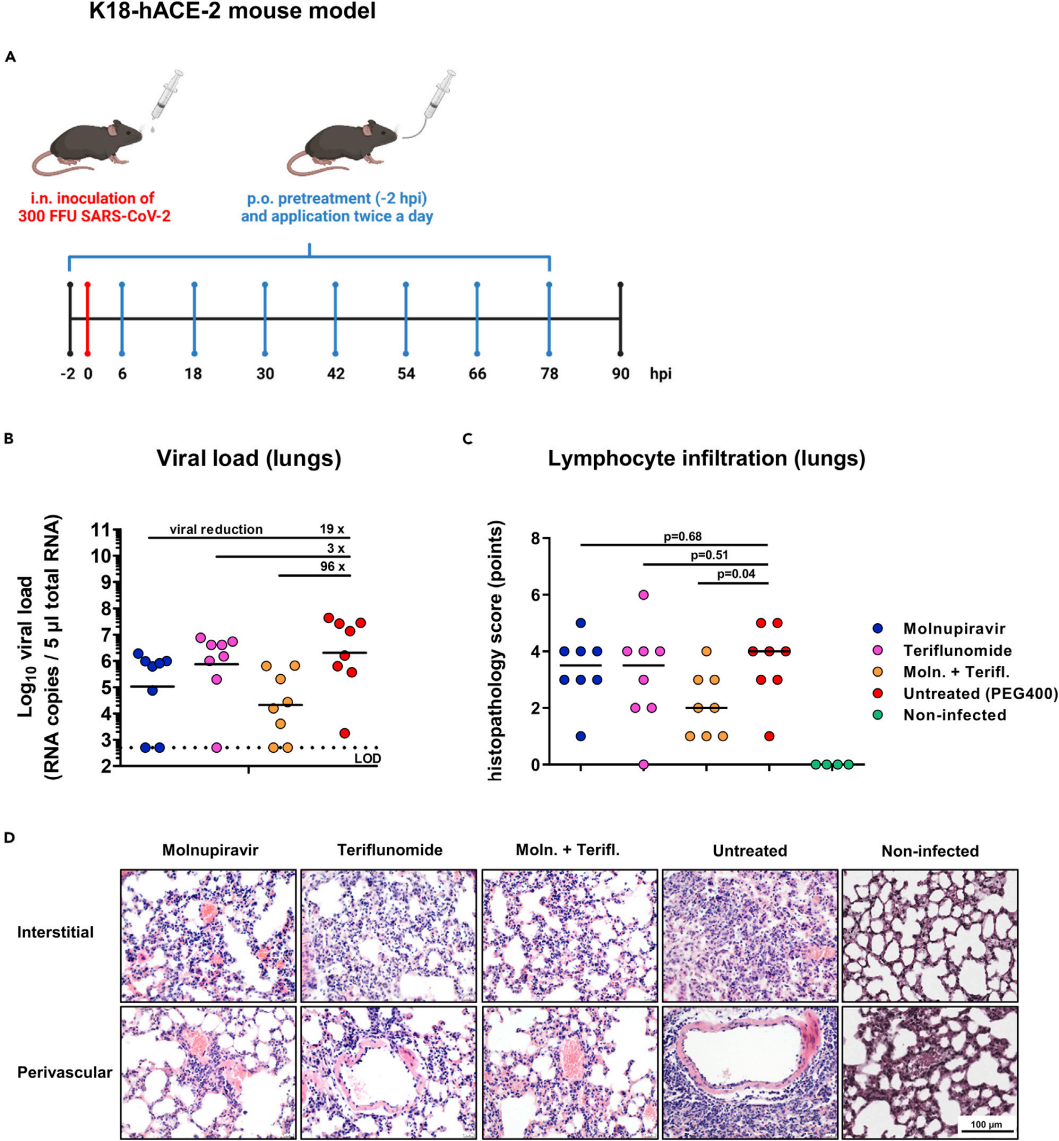
DHODH inhibitors cooperate with molnupiravir for treating COVID-19 in K18-hACE-2 mice (A) Treatment and infection scheme (drawn with BioRender.com). Female K18-hACE-2 mice (n = 8) received either 50 mg/kg bid molnupiravir, 10 mg/kg/day teriflunomide, or a mixture of molnupiravir + teriflunomide via oral gavage 2 h prior to infection with SARS-CoV-2, and 6 h thereafter. The drugs were then provided to the animals in a 12 h application cycle. The untreated control group received the vehicle solution PEG400. (B) Reduced SARS-CoV-2 RNA load in the lungs of mice upon combinatory treatment with molnupiravir and DHODH inhibitors. Viral RNA was isolated from lung homogenates and quantified by qRT-PCR 4 days after infection. Data points represent the viral RNA copy number found for each animal, along with the geometric mean of each group (n = 8). Reduction in viral load is shown as fold reduction compared to the untreated control. Statistical evaluation was performed using the Mann-Whitney U test. For p values, see Figure S7B. (C) Reduced lymphocyte infiltration of the lungs of SARS-CoV-2-infected mice upon treatment with molnupiravir and DHODH inhibitors. Female K18-hACE-2 mice (n = 8) were treated and infected as in (A), followed by histopathological analysis of lungs (H&E stains). For comparison, lungs from non-infected mice of the same genotype, taken from a different series of experiments, were investigated. The infiltration of the lungs with perivascular and interstitial lymphocytes was first scored separately (0–3), and scores were summed up for each animal. The scores (points) for each animal are depicted, along with the median of each group (n = 8). Statistical evaluation was performed by the Mann-Whitney U test. For p values, see Figure S7C. (D) Representative images of lymphocyte infiltration, as described in (C). Images of non-infected lung tissue from K18-hACE-2 mice were taken from a different series (historical control) and added to the panel. Bar, 100 μm.

Finally, we assessed the efficacy of the drug combination on virus replication and lung pathology in transgenic mice expressing human ACE2, the receptor of SARS-CoV-2, under the control of the Keratin K18 promoter (McCray et al., 2007; Oladunni et al., 2020), as we described previously (Peter et al., 2021). Before and during infection, mice were treated orally with the DHODH inhibitor teriflunomide and/or molnupiravir, with a similar schedule as in the hamster experiments (Figure 8A). Disease progression in this model was fast, leaving little change in animal weight (Figure S7A) and requiring termination of the experiment at day 4 p.i.. Notably, the combination of both drugs reduced the virus titer in the lungs of the animals, by a factor of up to 96-fold (Figures 8B and S7B). Moreover, lymphocyte infiltrations in the lungs of these animals were reduced accordingly (Figures 8C, 8D, and S7C). This transgenic mouse model involves virus-induced encephalitis (McCray et al., 2007; Sun et al., 2020). This was reflected by substantial virus load in the brain of infected mice. Interestingly, each of the drugs suppressed detectable virus in the brains of the animals (Figure S7D). Of note, the synergy of the drugs found *in vitro* was not recapitulated in a statistically significant fashion in the mice, neither in the lungs nor in the brains. We did find a trend toward higher efficiency of the drug combination regarding the virus load and lymphocyte infiltration within the lungs of the animals. Taken together, combining molnupiravir with a DHODH inhibitor was not overtly synergistic but appears as a promising strategy even in this highly susceptible animal model of SARS-CoV-2 infection.

## Discussion

Our results demonstrate that the simultaneous application of NHC and DHODH inhibitors suppresses the replication of SARS-CoV-2 in cultured cells far more profoundly than treatment with single drugs. The combination is also effective in animal models. Because both classes of compounds are singularly undergoing advanced clinical evaluation for the treatment of COVID-19, our data raise the perspective of using both drugs together as an antiviral combination therapy.

When using cell lines as an infection model, the two drugs displayed strong degrees of statistically significant synergies (Figure 2). The results of *in vivo* models, while still revealing the strongest effects when putting both drugs together, did not fulfill the strict criteria of synergism. Perhaps, the uridine in the serum of the animals partially compensates the effects of the DHODH inhibitors. Higher doses of DHODH inhibitors might possibly overcome this in future studies.

Besides their cooperation, another advantage of both NHC and DHODH inhibitors is their robustness toward virus variants, as exemplified (Figure 4). This was expected since neither of the drugs works by inhibiting a viral enzyme. DHODH inhibitors target a cellular, not a viral function, i.e. pyrimidine synthesis; NHC, in turn, works through incorrect base pairing, not by inhibition of viral enzymes. Indeed, even prolonged NHC treatment did not lead to resistance formation in other coronaviruses (Agostini et al., 2019). This raises the expectation that even novel virus variants, e.g. Omicron, remain susceptible to the drug combination.

On top of enhancing the incorporation of NHC into viral RNA, DHODH inhibitors also induce metabolic stress signaling, leading to the induction of an innate immune response independent of type I interferons. In addition, owing to their selective inhibition of hyperactive immune cells and excessive cytokine production, they may reduce the hyperinflammation, termed “cytokine storm” by some authors (Fajgenbaum and June, 2020), in late stage COVID-19. This can be beneficial, as exemplified by the successful treatment of COVID-19 patients with the steroid dexamethasone (Horby et al., 2021; Tomazini et al., 2020). In fact, DHODH inhibitors are currently in clinical use to treat autoimmune diseases like multiple sclerosis and rheumatoid arthritis (Munier-Lehmann et al., 2013), further supporting their usefulness to dampen excessive immune responses. In summary, we propose that the combination of DHODH inhibitors with NHC targets and abolishes virus replication, whereas DHODH inhibition may also ameliorate COVID-19-associated immunopathology.

It remains to be determined how DHODH inhibitors will affect the T cell response in the context of COVID-19. In general, DHODH inhibition interferes with T cell activity and thus suppresses the immune response (Fragoso and Brooks, 2015). However, the T cell response in the context of COVID-19 is unusual, with CD16-positive T cells mediating excessive cytotoxicity (Georg et al., 2022). It is therefore difficult to predict whether interfering with T cell proliferation will be beneficial when simultaneously counteracting virus replication.

Mechanistically, it is conceivable that reduced intracellular levels of cytidine triphosphate (resulting from DHODH inhibition) attenuate the competition and thus enhance the use of triphosphorylated NHC for incorporation into nascent virus RNA, i.e. both genomic and mRNA (Figure 1A). This was further corroborated by the rescue of virus replication by uridine and cytidine (Figure 5), each metabolic precursors of CTP. NHC triphosphate is generated by the salvage pathway for pyrimidines but not by *de novo* pyrimidine synthesis, suggesting that the levels of NHC triphosphate are not impaired by DHODH inhibition. Thus, upon combined treatment, we propose that virus RNA will contain a larger proportion of NHC versus cytidine. In subsequent rounds of virus RNA replication, this will lead to misincorporations of adenine bases instead of guanine (Gordon et al., 2021; Janion, 1978; Janion and Glickman, 1980; Kabinger et al., 2021; Salganik et al., 1973) with higher frequency, and thereby render the virus genome nonfunctional due to missense and nonsense mutations.

Previously (Stegmann et al., 2021a), we have established Methotrexate, a suppressor of purine biosynthesis, as an antagonist to SARS-CoV-2 replication, and this has been confirmed and expanded (Caruso et al., 2021; Zhang et al., 2021). The principle behind this approach is similar to that of DHODH inhibitors, which also interfere with nucleotide biosynthesis.

It remains to be determined how broadly the concept of combining DHODH inhibitors with pyrimidine-based antivirals is applicable. Molnupiravir was initially developed to counteract influenza virus replication (Toots et al., 2019, 2020). Even earlier, NHC was found to antagonize the propagation of coronavirus NL63 (Pyrc et al., 2006). Other reports describe the impact of NHC on the replication of bovine viral diarrhea and hepatitis C virus (Stuyver et al., 2003), norovirus (Costantini et al., 2012), Ebola virus (Reynard et al., 2015), Chikungunya virus (Ehteshami et al., 2017), respiratory syncytial virus (Yoon et al., 2018), SARS-CoV (Barnard et al., 2004), newly emerging variants of SARS-CoV-2 (Abdelnabi et al., 2021), and seasonal coronaviruses (Wang et al., 2021b). Viruses susceptible to DHODH inhibition were negative-sense RNA viruses (Influenza A and B Viruses, Newcastle Disease Virus, Vesicular Stomatitis Virus), positive-sense RNA viruses (Sindbis Virus, Hepatitis C virus, West Nile Virus, and Dengue Virus), DNA viruses (Vaccinia Virus and human Adenovirus), Mammarenaviruses (Kim et al., 2020), and HIV (Hoffmann et al., 2011). The combination of NHC and DHODH inhibitors might thus be suitable to inhibit the propagation of a broad range of viruses. This spectrum might be even further increased by other pyrimidine-based antivirals, e.g. Sofosbuvir in treating hepatitis C virus infection (Manns et al., 2017), Lamivudine and Telbivudine against hepatitis B virus (Yuen et al., 2018), Brivudine to counteract varicella zoster virus (De Clercq, 2004), as well as several pyrimidine-based drugs against HIV (Deeks et al., 2015).

NHC is a mutagen to bacteria (Janion, 1978; Janion and Glickman, 1980; Jena, 2020; Negishi et al., 1983; Popowska and Janion, 1974; Salganik et al., 1973). Presumably, NHC is converted to its 2′-deoxy form and then incorporated into bacterial DNA, followed by false incorporation of adenine bases in the following rounds of DNA replication. It remains to be explored how this will affect the pulmonary or intestinal microbiome in patients with COVID-19. Moreover, it is currently unclear to what extent NHC induces mutagenesis in mammalian cells and possibly in patients when treated with its prodrug molnupiravir, and this raised concerns that it might be carcinogenic and/or teratogenic. A moderate level of mutations in the gene HPRT1 was found after prolonged incubation of cultured cells with NHC (Zhou et al., 2021), but it is unclear whether this might translate into relevant mutagenesis in patients. When used for a few days, as recommended, molnupiravir has not been reported to cause any unacceptable levels of toxicity so far in the target population (adult, contraception, non-pregnant) (Khoo et al., 2021; Painter et al., 2021), although long-term follow-up is pending. Remarkably, ribavirin was not found carcinogenic after being used for decades in hepatitis C treatment, although at least one of its mechanisms of action is also based on mutagenesis of virus RNA (Crotty et al., 2000; Testoni et al., 2014). Thus, there is reason for justifying the cautious use of NHC-based drugs, while prohibiting their use in individuals who are trying to become pregnant or during pregnancy.

Another concern consists in the possible occurrence of transmissible virus mutants/variants from patients treated with molnupiravir. This possibility argues in favor of a “hit hard and early” combination treatment strategy and sufficiently long treatment to eradicate the virus—similar to most antibiotic regimens for treating bacterial infections.

Remarkably, NHC treatment of coronavirus-infected cells did not give rise to NHC-resistant viruses, even after prolonged and repeated incubation (Agostini et al., 2019). Likewise, DHODH-inhibitors have a cellular target that does not directly interact with a viral factor, thus providing little if any opportunity for resistant virus mutants to arise. Along with our finding that current virus variants of concern (VOC) respond similarly to the original SARS-CoV-2 strain (Figure 4), this raises the hope that the drug combination will be universally applicable to treat most if not all SARS-CoV-2 variants.

## STAR★Methods

### Key resources table

**Table:** 

REAGENT or RESOURCE	SOURCE	IDENTIFIER
**Antibodies**		

SARS-CoV-2 Spike	GeneTex	Cat# 632604; RRID: AB_2864418
SARS-CoV-2 Nucleoprotein	Sino Biological	Cat# 40143-R019; RRID: AB_2827973
DHODH	Santa Cruz	Cat# sc-166348; RRID: AB_2091729
HSC70	Santa Cruz	Cat# sc7298; RRID: AB_627761
J2/dsRNA:	Millipore	Cat# MABE1134; RRID: AB_2819101
Alexa Fluor 488 donkey anti-mouse	Thermo Fisher Scientific	Cat# A21202; RRID: AB_141607
Alexa Fluor 488 goat anti-mouse	Thermo Fisher Scientific	Cat# A11001; RRID: AB_2534069
Alexa Flour 546 donkey anti-rabbit	Thermo Fisher Scientific	Cat# A10040; RRID: AB_2534016
Donkey anti-mouse IgG, HRP conj.	Jackson ImmunoResearch	Cat# 715036,150; RRID: AB_2340773
Donkey anti-rabbit IgG, HRP conj.	Jackson ImmunoResearch	Cat# 711036152; RRID: AB_2340590

**Bacterial and virus strains**		

SARS-CoV-2 ‘wildtype’, Göttingen/Germany	Isolated from patient (Stegmann et al., 2021a)	N/A
hCoV-19/Germany/BY-Bochum-1/2020	Isolated from patient (Meister et al., 2021)	GISAID: EPI_ISL_1118929
SARS-CoV-2 Germany/BavPat1/2020 (BavPat1)	Friedrich Loeffler Institute (Germany)	GISAID: EPI_ISL_406862
SARS-CoV-2 Alpha (B.1.1.7)	Robert Koch Institute (Berlin, Germany)	GISAID: EPI_ISL_751799
SARS-CoV-2 Beta (B.1.351)	Robert Koch Institute (Berlin, Germany)	GISAID: EPI_ISL_803957
SARS-CoV-2 Delta (B.1.617.2)	Robert Koch Institute (Berlin, Germany)	GISAID: EPI_ISL_2378732

**Chemicals, peptides, and recombinant proteins**		

β-D-N^4^-Hydroxycytidine (NHC/EIDD-1931)	Cayman Chemical	Cat# 9002958
IMU-838	Immunic Therapeutics	N/A
BAY2402234	Selleckchem	Cat# S8847
Teriflunomide	Selleckchem	Cat# S4169
Teriflunomide	Biozol	Cat# CBS-FT16895
ASLAN003	Selleckchem	Cat# S9721
Brequinar	Selleckchem	Cat# S6626
Uridine	Selleckchem	Cat# S2029
Cytidine	Selleckchem	Cat# S2053
Molnupiravir	MedChemExpress LLC	Cat# HY-135853
DMSO	Applichem	Cat# A3672.0100
Crystal violet	Roth	Cat# T123.1
Methanol	Roth	Cat# 8388.3
Lysis Binding buffer (from MagNA Pure LC Total Nucleic Acid Isolation Kit)	Roche	Cat# 03038505001
Trizol LS	Life Technologies	Cat# 10296028
Trichlormethan/Chloroform	Applichem	Cat# 3313.1
Isopropanol	Applichem	Cat# 6752.2
Ethanol	ChemSolute/Th.Geyer	Cat# 11647081/2246
Triton X-100	Applichem	Cat# A1388
Nuclease free water	Ambion	Cat# AM9939
PEG400	Roth	Cat# 0144.1
Tween-80	Sigma Aldrich	Cat# P4780
Peanut Oil	Braendle	Cat# 913 or EAN# 4,008,722,009,130
Strawberry Syrup	Yo	EAN# 9001400005009
4′,6-Diamidino-2- Phenylindole (DAPI)	Sigma	Cat# D9542-5MG
Fluorescence Mounting Medium (DAKO)	DakoCytomation	Cat# S302380-2
Shandon Immu-Mount	Thermo Fisher Scientific	Cat# 9990402
Hoechst33342	Thermo Fisher Scientific	Cat# H3570
Formalin	Roth	Cat# 7398.1
Paraffin	VWR	Cat# 1071642504
Hematoxylin	Roth	Cat# 3816.1
Eosin (HE)	Th. Geyer, RV	Cat# E6003-25G

**Critical commercial assays**		

Pierce BCA Protein Assay Kit	Thermo Fisher Scientific	Cat# 23227
Immobilon Western Substrate	Millipore	Cat# WBKLS0500
Super Signal West Femto Maximum Sensitivity Substrate	Thermo Fisher Scientific	Cat# 34095
LDH-Glo^TM^ Cytotoxicity Assay kit	Promega	Cat# J2380
CellTiter-Glo® Luminescent Cell Viability Assay	Promega	Cat# G7570
StemDiff™ Branching Lung Organoid Kit	Stemcell	Cat# 100-0195
NucleoMag®VET kit	Machery & Nagel GmbH	Cat# 744200.4
QIAamp Viral RNA Mini Kit	Qiagen	Cat# 52904
TaqMan® Fast Virus 1-Step Master Mix	Thermo Fisher Scientific	Cat# 4444432
AgPath-ID One-Step RT-PCR Kit	Applied Biosystems	Cat# AM1005

**Experimental models: Cell lines**		

*Monkey*: Vero E6 (Vero C1008)	ATCC	Cat# CRL-1586
*Human*: Calu-3	ATCC	Cat# HTB55
CD34-positive human induced pluripotent stem cells (hiPSCs)	Dorn et al. (2015)	N/A

**Experimental models: Organisms/strains**		

Syrian Gold hamsters	Janvier Labs, Saint Berthevin, France	N/A
C57BL/6 mice	Janvier Labs, Saint Berthevin, France	N/A
K18-hACE-2 mice	Charles River Laboratories	704K18-HACE2

**Oligonucleotides**		

Primer (probe), with 5′FAM, 3′BBQ ACA CTA GCC ATC CTT ACT GCG CTT CG	Eurofins Genomics	N/A
Primer (forward) ACA GGT ACG TTA ATA GTT AAT AGC GT	Eurofins Genomics	N/A
Primer (reverse) ATA TTG CAG CAG TAC GCA CAC A	Eurofins Genomics	N/A

**Software and algorithms**		

Prism (version 9.0.0)	GraphPad	N/A
SynergyFinder	Zheng et al. (2022)	https://synergyfinder.org
AxioVision Zen 2 (2.0.0.0)	Zeiss	N/A
Image Lab™ (version 5.2.1)	Bio-Rad	N/A
BioRender	BioRender	https://biorender.com/
SPSS (version 20.0)	SPSS Inc.	N/A
NDPview.2 plus (version 2.8.24)	Hamamatsu Photonics, K.K. Japan	N/A

### Resource availability

#### Lead contact

Further information and requests for resources and reagents should be directed to and will be fulfilled by the lead contact, Matthias Dobbelstein (mdobbel@uni-goettingen.de).

#### Materials availability

This study did not generate new unique reagents.

### Experimental model and subject details

Vero E6 cells (Vero C1008) and Calu-3 cells were obtained from the German Primate Research Center Göttingen.

Lung organoids were generated from CD34-positive human induced pluripotent stem cells (hiPSCs) using a modified StemDiff™ Branching Lung Organoid Kit (Stemcell™) protocol.

Eight-week-old Syrian Gold hamsters (72–96 g) were obtained from Janvier Labs (Saint Berthevin, France).

K18-hACE-2 mice were from Jackson Laboratories (USA).

### Method details

These sections are combined for better readability, since the methods and analyses were different for each experimental model system.

#### SARS-CoV-2 replication in Vero E6 and Calu-3 cells

##### Cell culture

Vero E6 cells (Vero C1008) were maintained in Dulbecco’s modified Eagle’s medium (DMEM with GlutaMAX^TM^, Gibco) supplemented with 10% fetal bovine serum (Merck), 50 units/mL penicillin, 50 μg/mL streptomycin (Gibco), 2 μg/mL tetracycline (Sigma) and 10 μg/mL ciprofloxacin (Bayer) at 37°C in a humidified atmosphere with 5% CO_2_. Calu-3 cells were maintained in Eagle’s Minimum Essential Medium (EMEM, ATCC) supplemented with 10% fetal bovine serum and penicillin/streptomycin.

##### Treatments, SARS-CoV-2 infection and TCID_50_ determination

30,000 cells per well were seeded into 24-well-plates using medium containing 2% fetal bovine serum (FBS) and incubated for 8 h at 37°C. Cells were treated with β-D-N^4^-Hydroxycytidine (NHC/EIDD-1931, Cayman Chemical 9002958), IMU-838 (Immunic Therapeutics), BAY2402234 (Selleckchem S8847), Teriflunomide (Selleckchem S4169), ASLAN003 (Selleckchem S9721), Brequinar (Selleckchem S6626), Uridine (Selleckchem S2029) or Cytidine (Selleckchem S2053) at the concentrations indicated in the figure legends. When preparing stock solutions, all compounds were dissolved in DMSO. After 24 h, cells were infected with virus stocks corresponding to 1∗10^7^ RNA-copies of SARS-CoV-2 (= 30 FFU) and incubated for 48 h at 37°C, as described (Stegmann et al., 2021a). The original SARS-CoV-2 ‘wildtype’ was isolated from a patient sample taken in March 2020 in Göttingen, Germany (Stegmann et al., 2021a). The SARS CoV-2 variants hCoV-19/Germany/BY-Bochum-1/2020 (GISAID accession ID: EPI_ISL_1118929), Alpha (B.1.1.7, EPI_ISL_751799), Beta (B.1.351, EPI_ISL_803957) and Delta (B.1.617.2, EPI_ISL_2378732) were kindly provided by the Robert Koch Institute, Berlin, Germany.

To determine the Median Tissue Culture Infectious Dose (TCID_50_) per mL, 30,000 Vero E6 cells per well were pre-treated with NHC, IMU-838 or the indicated combinations for 24 h, infected with SARS-CoV-2 strains (MOI 0.1) for 1 h and after washing again treated with the drugs for additional 48 h. The virus-containing supernatant was titrated (endpoint dilution assay) to calculate the TCID_50_/mL. Vero E6 cells were incubated with 10-fold dilutions (quadruplicates) of virus for 4 days and then incubated with 0.1% crystal violet in 20% (v/v) methanol. The titer was determined according to Spearman and Kärber (1931).

##### Quantitative RT-PCR for virus quantification

For RNA isolation, the SARS-CoV-2-containing cell culture supernatant was mixed (1:1 ratio) with the Lysis Binding Buffer from the MagNA Pure LC Total Nucleic Acid Isolation Kit (Roche) to inactivate the virus. The viral RNA was isolated using Trizol LS, chloroform, and isopropanol. After washing the RNA pellet with ethanol, the isolated RNA was re-suspended in nuclease-free water. Quantitative RT-PCR was performed according to a previously established RT-PCR assay involving a TaqMan probe (Corman et al., 2020), to quantify virus RNA yield. The following oligonucleotides were used for qRT-PCR, which amplify a genomic region corresponding to the envelope protein gene (26,141–26,253), as described (Corman et al., 2020).

**Table:** 

Primer	Sequence	Modification
P (probe)	ACA CTA GCC ATC CTT ACT GCG CTT CG	5′FAM, 3′BBQ
F (forward)	ACA GGT ACG TTA ATA GTT AAT AGC GT	
R (reverse)	ATA TTG CAG CAG TAC GCA CAC A	

The amount of SARS-CoV-2 RNA determined upon infection without any treatment was defined as 100%, and the other RNA quantities were normalized accordingly.

##### Determination of synergy between drug combinations

Vero E6 cells were treated/infected as indicated and RNA within the cell culture supernatant was isolated and quantified by qRT-PCR. The amount of SARS-CoV-2 RNA determined upon infection without any treatment was defined as 100% virus yield (0% inhibition). The other samples were normalized accordingly and stated as percentage of control. The Bliss independence model (Bliss, 1939), calculated using the synergyfinder (Ianevski et al., 2020) at https://synergyfinder.org/was used to quantify synergy between drug combinations. We did not assume mutual exclusivity of the drug effects.

##### Immunofluorescence analyses

Vero E6 cells were seeded onto 8-well chamber slides (Nunc) and treated/infected as indicated. After 48 h of SARS-CoV-2 infection, the cells were washed once in PBS and fixed with 4% formaldehyde in PBS for 1 h at room temperature. After permeabilization with 0.5% Triton X-100 in PBS for 30 min and blocking in 10% FBS/PBS for 10 min, primary antibodies were used to stain the SARS-CoV-2 Nucleoprotein (N; Sino Biological #40143-R019, 1:8000) and Spike protein (S; GeneTex#GTX 632604, 1:2000) overnight. The secondary Alexa Fluor 546 donkey anti-rabbit IgG and Alexa Fluor 488 donkey anti-mouse IgG (Invitrogen, 1:500, diluted in blocking solution) antibodies were added together with 4′,6-diamidino-2-phenylindole (DAPI) for 1.5 h at room temperature. Slides with cells were mounted with Fluorescence Mounting Medium (DAKO) and fluorescence signals were detected by microscopy (Zeiss Axio Scope.A1).

##### Immunoblot analysis

Cells were washed once in PBS and harvested in radioimmunoprecipitation assay (RIPA) lysis buffer (20 mM TRIS-HCl pH 7.5, 150 mM NaCl, 10 mM EDTA, 1% Triton X-100, 1% deoxycholate salt, 0.1% SDS, 2 M urea), supplemented with protease inhibitors. Samples were briefly sonicated and protein extracts quantified using the Pierce BCA Protein assay kit (Thermo Fisher Scientific). After equalizing the amounts of protein, samples were incubated at 95°C in Laemmli buffer for 5 min and separated by SDS PAGE (SDS-PAGE). To determine the presence of viral proteins, the separated proteins were transferred to a nitrocellulose membrane, blocked in 5% (w/v) non-fat milk in TBS containing 0.1% Tween 20 for 1 h, and incubated with primary antibodies at 4°C overnight, followed by incubation with peroxidase-conjugated secondary antibodies (donkey anti-rabbit or donkey anti-mouse IgG, Jackson Immunoresearch). The SARS-CoV-2 Spike- and Nucleoprotein, DHODH and HSC70 (loading control) were detected using either Super Signal West Femto Maximum Sensitivity Substrate (Thermo Fisher) or Immobilon Western Substrate (Millipore).

**Table:** 

Antibody	Source (Catalog number)	Dilution
SARS-CoV-2 Spike	GeneTex GTX 632604	1:1,000
SARS-CoV-2 Nucleoprotein	Sino Biological 40143-R019	1:5,000
DHODH	Santa Cruz sc-166348	1:250
HSC70	Santa Cruz sc-7298	1:10,000

##### Quantification of LDH release to determine cytotoxicity

3,500 Vero E6 cells were seeded into 96-well-plates and treated with DHODH inhibitors and/or NHC as indicated, for 72 h (corresponding to the incubation of cells with drugs before and after infection). The release of lactate dehydrogenase (LDH) into the cell culture medium was quantified by bioluminescence using the LDH-Glo^TM^ Cytotoxicity Assay kit (Promega). 10% Triton X-100 was added to untreated cells for 15 min to determine the maximum LDH release, whereas the medium background (= no-cell control) served as a negative control. Percent cytotoxicity reflects the proportion of LDH released to the media compared to the overall amount of LDH in the cells, and was calculated using the following formula.

$$\text{Cytotoxicity }\left(\text{\%}\right)=100\times \frac{\left(\text{Experimental}\;\text{LDH}\;\text{Release}-\text{Medium}\;\text{Background}\right)\;}{\left(\text{Maximum}\;\text{LDH}\;\text{Release}\;\text{Control}-\text{Medium}\;\text{Background}\right)}$$

##### Quantification of ATP to determine cell viability

3,500 Vero E6 cells were seeded into 96-well plates and treated with DHODH inhibitors and/or NHC as indicated. After 72 h, the CellTiter-Glo® Luminescent Cell Viability Assay solution (Promega) was added to the cell culture supernatant (1:1 ratio) for 10 min, followed by luminometry using a Centro LB 960 luminometer (Berthold). The extent of luminescence, reflecting relative ATP levels, was normalized to DMSO-treated cells.

#### SARS-CoV-2 replication in lung organoids

##### Human lung organoids

Lung organoids were generated from CD34-positive human induced pluripotent stem cells (hiPSCs) using a modified StemDiff™ Branching Lung Organoid Kit (Stemcell™) protocol. 120 days-old organoids were sliced using a Leica VT 100 S vibrating microtome (Leica) and treated with β-D-N4-Hydroxycytidine and/or BAY2402234. After 24 h, the organoids were infected with 35,000 PFU per slice of the original SARS-CoV-2 strain (GISAID accession ID: EPI_ISL_1118929) for 2 h. After three washes, medium containing the indicated compound concentrations was added. Viral titers were determined by limited dilution assay as TCID_50_/mL and calculated with the Spearman-Kärber method 24, 48, and 72 h post infection. The organoids, fixed in 4% PFA, were frozen in a 1:1 mixture of 30% (w/v) sucrose in PBS and O.C.T. tissue freezing medium (Sakura). For immunofluorescence analysis, samples were sliced into 16 μm sections, permeabilized with 0.25% Triton X-100, and blocked with 0.1% Triton X-100 and 5% normal goat serum in PBS. The primary antibody (J2/dsRNA: MABE1134, Merck Millipore, 1:400) and secondary antibody (goat anti mouse IgG Alexa 488: A-11001, Thermo Fisher Scientific, 1:1000) were each diluted in 0.1% Triton X-100 in PBS. Nuclei were stained with 0.001 mg/mL Hoechst33342 (Thermo Fisher Scientific) in ddH_2_O. Samples were mounted with Shandon Immu-Mount (Thermo Fisher Scientific). Immunohistochemically stained samples were imaged with a Leica TCS SP8 confocal microscope.

#### Pharmacokinetics (PK) of Molnupiravir and Teriflunomide in hamsters and mice

##### Ethics statement

The experiments were carried out according to the German Regulations for Animal Welfare after obtaining the necessary approval from the ethics committee authorized by Regierungspräsidium Tübingen. Experiments were performed at Synovo GmbH, Tübingen, Germany.

##### PK experiment in hamsters

Eight-week-old Syrian Gold hamsters (72–96 g) were obtained from Janvier Labs (Saint Berthevin, France). The animals received either 150 mg/kg Molnupiravir (MedChemExpress LLC) twice daily or 20 mg/kg/day Teriflunomide (Biozol) via oral application using a feeding pipet (n = 3 per group). Teriflunomide was formulated in 20% PEG400 (Roth), 2% Tween-80 (Sigma Aldrich), 39% peanut oil (Braendle) and 39% strawberry syrup (Yo); Molnupiravir was formulated in 10% PEG400, 2% Tween-80, 44% water and 44% strawberry syrup. Molnupiravir was given twice daily with 8 h between first and second treatment each day in an application volume of 4 mL/kg. Formulations were freshly prepared before every application. At day one, PK samples were taken 0.5, 1, 2, 4, 8 and 24 h after the first dose. Compound concentrations were analyzed in whole blood using liquid chromatography-mass spectrometry. For Molnupiravir, its active metabolite, EIDD-1931/NHC, was measured.

##### PK experiment in mice

Eight-week-old female C57BL/6 mice (19–21 g) were obtained from Janvier Labs (Saint Berthevin, France). The animals received either 50 mg/kg Molnupiravir (MedChemExpress LLC) twice daily, 10 mg/kg/day Teriflunomide (Biozol) or a mixture of 50 mg/kg twice daily Molnupiravir +10 mg/kg/day Teriflunomide via oral gavage in a volume of 2.5 mL/kg PEG400 (n = 3 per group). Molnupiravir was given twice daily with 8 h between first and second treatment each day. Formulations were freshly prepared before every application. At day one, PK samples were taken 0.5, 1, 2, 4, 6 and 8 h after the first dose. Compound concentrations were analyzed as in the hamster PK experiment.

#### Modeling COVID-19 in Syrian Gold hamsters

##### Ethics statement

Hamster experiments were carried out according to the German Regulations for Animal Welfare after obtaining the necessary approval from the authorized ethics committee of the State Office of Agriculture, Food Safety and Fishery in Mecklenburg – Western Pomerania (LALLF M−V) under permission number 7221.3-1-049/20 and approval of the commissioner for animal welfare at the Friedrich Loeffler Institute (FLI), representing the Institutional Animal Care and Use Committee (IACUC).

##### Hamster experiment

Male Syrian Gold hamsters (80–100 g) were obtained from Janvier Labs (Saint Berthevin, France). Hamster experiments were conducted in BSL 3 animal facilities. Four hamsters were housed in individually ventilated cages per group. Hamsters were inoculated by the orotracheal route with 1∗10^4^ TCID_50_ SARS-CoV-2 Germany/BavPat1/2020 (BavPat1) (GISAID accession EPI_ISL_406862) in a volume of 100 μL. 250 mg/kg Molnupiravir alone, 10 mg/kg Teriflunomide alone, or the combination of both were administered twice a day with 6–8 h in between, starting 24 h before inoculation until six days post inoculation. Body weight was documented daily for seven days. Nose fluid samples were collected at day two and four post inoculation and were serially diluted and titrated according to the description above (TCID_50_). At day 7 post inoculation, the animals were sacrificed and a full autopsy was performed under BSL3 conditions. The treatment scheme was depicted using BioRender.com.

##### Detection of virus RNA

Extraction of total RNA from nose fluid samples from hamsters was done using the “Viral RNA/DNA isolation” NucleoMag®VET kit (Machery & Nagel GmbH, Düren, Germany) in a KingFisher Flex Purification System (ThermoFisher Scientific, USA). SARS-CoV-2 RNA was detected using the AgPath-ID One-Step RT-PCR Kit (E-gene Sarbeco 6-carboxyfluorescein quantitative RT-PCR) (Applied Biosystems) as published previously (Corman et al., 2020).

##### Determination of lung affection by histopathology

During the necropsy, the percentage of dark red discoloration per total lung tissue was estimated. The left lung lobe was carefully removed, immersion-fixed in 10% neutral-buffered formalin, and paraffin-embedded. 2-3-μm-thick sections were stained with hematoxylin and eosin (HE). Slides were scanned using a Hamamatsu S60 scanner, and the evaluation was performed using the NDPview.2 plus software (Version 2.8.24, Hamamatsu Photonics, K.K. Japan). For histopathology, the left lung lobe was evaluated using a 500 × 500μm grid. The extent of pneumonia-associated consolidation was recorded as percentage of affected lung fields. Further, the lung was examined for the presence of SARS-CoV2-characteristic lesions described for hamsters, i.e. intra-alveolar, interstitial, peribronchial and perivascular inflammatory infiltrates, alveolar edema, necrosis of the bronchial and alveolar epithelium, diffuse alveolar damage, vasculitis or endothelialitis, pneumocyte type 2 hyperplasia/hypertrophy with atypical cells and hypertrophy/hyperplasia of the bronchial epithelium. Evaluation and interpretation was performed by a board-certified pathologist (DiplECVP) following a post examination masking approach (Meyerholz and Beck, 2018).

##### Determination of daily energy expenditure

The daily energy expenditure (DEE) was determined individually for nine hamsters (three not infected, three SARS-CoV-2 infected receiving no medication, three SARS-CoV-2 infected receiving Teriflunomid + Molnupiravir) for a total of four days using the doubly labeled water (DLW) method (Lifson and McClintock, 1966; Speakman, 1997), as explained in detail elsewhere (Riek et al., 2021). Briefly, hamsters were injected intraperitoneally with 1.99 ± 0.03 g DLW per kg body mass, (65% ^18^O and 35% ^2^H; 99.90% purity). The individual dose for each hamster was determined prior to the injection according to its body mass. Subsequently, after a 1-h equilibration period, blood samples of 70–100 μL were drawn by puncturing the gingival venous plexus of each hamster at 1, 48 and 96 h after dosing to estimate the isotope elimination rates. Serum from the blood samples were stored at −20°C until determination of ^18^O and ^2^H enrichment. The DEE was calculated from carbon dioxide production using a single pool model as is appropriate for this size of animal (Speakman, 1993), with Equation 7.17 in (Speakman, 1997) and converted to energy expenditure assuming a respiration quotient of 0.85 and the Weir equation (Weir, 1949). Isotope analyses and calculations were done in a blinded fashion regarding the status of the animals.

#### Modeling COVID-19 in transgenic mice

##### Ethics statement

Mouse experiments were carried out according to the German Regulations for Animal Welfare after obtaining the necessary approval from the authorized ethics committee of the State Saxony under the permission number 25–5121/515/7 (TVV 06/21).

##### Mouse experiment

Female K18-hACE-2 mice (n = 8) received either 50 mg/kg twice per day (bid) Molnupiravir, 10 mg/kg/day Teriflunomide, or a mixture of both, each via oral gavage in a volume of 100 μL, 2 h prior to infection, followed by a 12h-application cycle starting 6 h after virus inoculation. The mock control group received 2.5 μL/kg of the vehicle solution PEG400. Mice were infected intranasally, under isoflurane anesthesia, with 300 FFU of SARS-CoV-2 (strain BavPat1/2020) in 50 μL total volume. At day 4 after virus inoculation, the mice were euthanized and organs were homogenized in 2 mL PBS. Viral RNA was isolated from lung homogenates and quantified by qRT-PCR. A low inoculum was used for greater sensitivity of the assay, since this puts a higher demand on virus replication. Moreover, a low inoculum is more representative of what most COVID-19 patients receive when they get accidentally infected. The treatment scheme was depicted using BioRender.com.

##### Detection of virus RNA

Viral RNA was isolated from 140 μL of homogenates using QIAamp Viral RNA Mini Kit (Qiagen). RT-qPCRs were performed using TaqMan Fast Virus 1-Step Master Mix (Thermo Fisher) and 5 μL of isolated RNA as a template, as described (Groß et al., 2020). Synthetic SARS-CoV-2 RNA was used as a quantitative standard to obtain viral copy numbers. Statistical evaluation of the data was performed by Mann-Whitney U test in comparison to the mock control and single treatments.

##### Determination of lung affection by histopathology

Evaluation of mouse pathology was performed in analogy to the hamster experiments, as described above. A historical control of a non-infected mouse (same genotype) was added at a later time. Lymphocyte infiltration was determined in the perivascular and the interstitial regions of the murine lungs and scored each on a scale between 0 and 3. The sum of these scores was calculated for each animal.

### Quantification and statistical analysis

#### Quantification and statistical analysis of cell-based experiments

Statistical testing was performed using Graph Pad Prism 9. Unless otherwise specified, a two-sided unpaired Student’s *t* test was calculated, and significance was assumed where p ≤ 0.05. Asterisks represent significance in the following way: ∗∗∗∗, p ≤ 0.0001; ∗∗∗, p ≤ 0.005; ∗∗, p ≤ 0.01; ∗, p ≤ 0.05.

#### Quantification and statistical analysis of animal experiments

For statistical analyses and graphical illustrations, GraphPad Prism version 9.0.0 (GraphPad Software, La Jolla, CA) and SPSS version 20.0 (SPSS Inc., Chicago, IL, United States) were used. Hamster body weight was statistically analyzed by one-way ANOVA followed by post hoc Tukey tests (p < 0.05). All other data were non-parametric and tested by Kruskal-Wallis test with Dunn’s correction.

## Data Availability

All data reported in this paper will be shared by the Lead contact upon request. This paper does not report original code. Any additional information required to reanalyze the data reported in this paper is available from the Lead contact upon request.
