# Supplementary material for: Inhibitors of dihydroorotate dehydrogenase cooperate with molnupiravir and N4-hydroxycytidine to suppress SARS-CoV-2 replication
Source: iScience. 2022 Apr 25;25(5):104293. doi: 10.1016/j.isci.2022.104293 (PMC9035612; doi:10.1016/j.isci.2022.104293)

iScience, Volume *25*

Supplemental information

Inhibitors of dihydroorotate dehydrogenase cooperate with molnupiravir and N4-hydroxycytidine to suppress SARS-CoV-2 replication

Kim M. Stegmann, Antje Dickmanns, Natalie Heinen, Claudia Blaurock, Tim Karrasch, Angele Breithaupt, Robert Klopfleisch, Nadja Uhlig, Valentina Eberlein, Leila Issmail, Simon T. Herrmann, Amelie Schreieck, Evelyn Peelen, Hella Kohlhof, Balal Sadeghi, Alexander Riek, John R. Speakman, Uwe Groß, Dirk Görlich, Daniel Vitt, Thorsten Müller, Thomas Grunwald, Stephanie Pfaender, Anne Balkema-Buschmann, and Matthias Dobbelstein

FIGURE S1: The combination of NHC and DHODH inhibitors strongly impairs SARS-CoV-2 replication without detectable cytotoxicity, Related to Fig. 1

(A) Lack of measurable cytotoxicity by NHC. Vero E6 cells were treated with increasing concentrations of NHC for 72 h. The release of lactate dehydrogenase (LDH) to the supernatant was quantified by bioluminescence and displayed as percentage of total cellular LDH, as a read-out for cytotoxicity (mean with SD, n=3).

(B) Lack of measurable cytotoxicity by IMU-838. The experiment was carried out as in A, using IMU-838 instead of NHC.

(C) The combination of NHC and DHODH inhibitors does not affect cell viability. Vero E6 cells were treated as in Fig. 1 and ATP levels were measured by the CellTiter-Glo® assay. The extent of luminescence, reflecting relative ATP levels, was normalized to untreated cells (mean with SD, n=3).

FIGURE S2: Strong synergism of NHC and DHODH inhibitors to diminish the release of SARS-CoV-2 RNA, Related to Fig. 2

(A) Vero E6 cells were treated with NHC and/or DHODH inhibitors at the same time as they were infected with SARS-CoV-2. Note that the reduction in virus RNA progeny was less pronounced than with pre-treatment, but still clearly observable after co-administration of virus and treatment (mean with SD, n=3).

(B) Statistical analysis of the data shown in (A) was performed by a two-sided unpaired Student's t test. P values comparing untreated and treated samples were summarized.

(C) Diminished virus RNA progeny by NHC and DHODH inhibitors even when added 4 h after SARS-CoV-2 infection. Statistical analysis of the data shown in Fig. 2B was performed as in (B).

FIGURE S3: The combination of NHC with DHODH inhibitors synergistically reduces the replication of the SARS-CoV-2 Delta variant, Related to Fig. 4

(A) Reduced virus RNA progeny in the presence of NHC and DHODH inhibitors upon infection with the SARS-CoV-2 Delta variant (B.1.617.2). Vero E6 cells were treated with drugs and/or infected as in Fig. 1, followed by quantitative detection of SARS-CoV-2 RNA in the cell supernatant. The combination of NHC and the DHODH inhibitors IMU-838, BAY2402234 or Teriflunomide strongly reduced the amount of SARS-CoV-2 Delta variant RNA released to the supernatant compared to single drug treatments (mean with SD, n=3).

(B) Statistical analysis of the data shown in (A) was performed by a two-sided unpaired Student's t test. p values comparing untreated and treated samples were summarized.

FIGURE S4: Uridine as well as cytidine rescue SARS-CoV-2 replication in the presence of NHC and DHODH inhibitors, Related to Fig. 5

(A) The antiviral effect of DHODH inhibitors combined with NHC can be reverted by uridine. Statistical analysis of the data shown in Fig. 5A was performed by a two-sided unpaired Student's t test. P values comparing untreated and treated samples were summarized.

(B) Restored SARS-CoV-2 replication by cytidine, in the presence of NHC and DHODH inhibitors. Statistical analysis of the data shown in Fig. 5B was performed as in (A).

FIGURE S5: DHODH inhibitors cooperate with Molnupiravir for treating COVID-19 in Syrian Gold hamsters, Related to Fig. 7

(A) Pharmacokinetics of Molnupiravir and Teriflunomide in hamsters and mice. Syrian Gold hamsters were treated with 150 mg/kg Molnupiravir twice daily or with 20 mg/kg/day Teriflunomide. C57BL/6 mice were treated with 50 mg/kg Molnupiravir twice daily, 10 mg/kg/day Teriflunomide, or a mixture of 50 mg/kg Molnupiravir twice daily + 10 mg/kg/day Teriflunomide. Whole blood samples were taken at the indicated time points after the first dose. Concentrations of the active metabolite of Molnupiravir,

EIDD-1931/NHC, or of Teriflunomide, were analyzed. The arrows reflect the time points of treatment. For Molnupiravir, the second treatment of the day was given after blood sampling at 8 hours.

(B) Male Syrian Gold hamsters were treated with 250 mg/kg Molnupiravir, 10 mg/kg/day Teriflunomide, or a combination of 250 mg/kg Molnupiravir with 10 mg/kg Teriflunomide, administered twice a day, starting 24 h before inoculation until six days post inoculation with $1*10^4$ $TCID_{50}$ SARS-CoV-2. Virus load within lung organs was determined by $TCID_{50}$.

(C) Decreased loss in body weight of SARS-CoV-2-infected hamsters in the presence of Molnupiravir and Teriflunomide. Statistical analysis of the data shown in Fig. 7C was performed by one-way ANOVA followed by post hoc Tukey tests ($P < 0.05$). P values comparing untreated and treated samples were summarized.

FIGURE S6: DHODH inhibitors cooperate with Molnupiravir regarding lung pathology in Syrian Gold hamsters, Related to Fig. 7

(A) Quantification of macroscopic lesions. Male Syrian Gold hamsters were treated and infected as in (B). The extent of dark red discoloration given in % per total lung was quantified (mean, n=4).

(B) Histopathology identified reduced pneumonia-associated consolidation in SARS-CoV-2 infected animals after treatment with Molnupiravir, Teriflunomide or in combination. The area affected by pneumonia-associated consolidation given in % per total lung was quantified (mean, n=4). Data were analyzed by Kruskal-Wallis test with Dunn's correction.

(C) Histopathology of pneumonia-associated consolidation after Mock or $1*10^4$ $TCID_{50}$ SARS-CoV-2 infection. (a) mock group, no treatment, (b) infected, no treatment, (c) infected, 250 mg/kg Molnupiravir treatment, (d) infected, 10 mg/kg/day Teriflunomide treatment, (e) infected, combined treatment with 250 mg/kg Molnupiravir and 10 mg/kg Teriflunomide. (a-e) HE staining. Bar, 2.5 mm.

(D) Daily energy expenditure. Upon infection and treatment of hamsters as in Fig. 7A, the daily energy expenditure (DEE) was determined using the doubly labeled water (DLW) method. This method was only affordable to us to include animals that were either infected without treatment, or infected with combined treatment, to show that the drug combination improves the health of infected animals regardless of the contribution from each drug.

FIGURE S7: DHODH inhibitors cooperate with Molnupiravir for treating COVID-19 in K18-hACE-2 mice, Related to Fig. 8

(A) Female K18-hACE-2 mice (n=8) were treated and infected as in Fig. 8A. All animals were monitored daily for body weight. Data are presented as means ± standard deviation (SD).

(B) Reduced SARS-CoV-2 RNA load in murine lungs upon combination treatment with Molnupiravir or Teriflunomide. Statistical analysis of the data shown in Fig. 8B was performed by Mann-Whitney U test. p values comparing untreated and treated samples were summarized.

(C) Reduced lymphocyte infiltration in murine lung tissue upon combination treatment with Molnupiravir and Teriflunomide. Statistical analysis of the data shown in Fig. 8C was performed as in (B).

(D) Female K18-h-ACE-2 mice (n=8) were treated and infected as in Fig. 8A. Viral RNA was isolated from brain homogenates and quantified by qRT-PCR 4 days after infection. Data points represent the viral RNA copy number found for each animal, along with the geometric mean of each group. Reduction in viral load is shown as fold reduction compared to the untreated control.

Figure S1, related to Figure 1

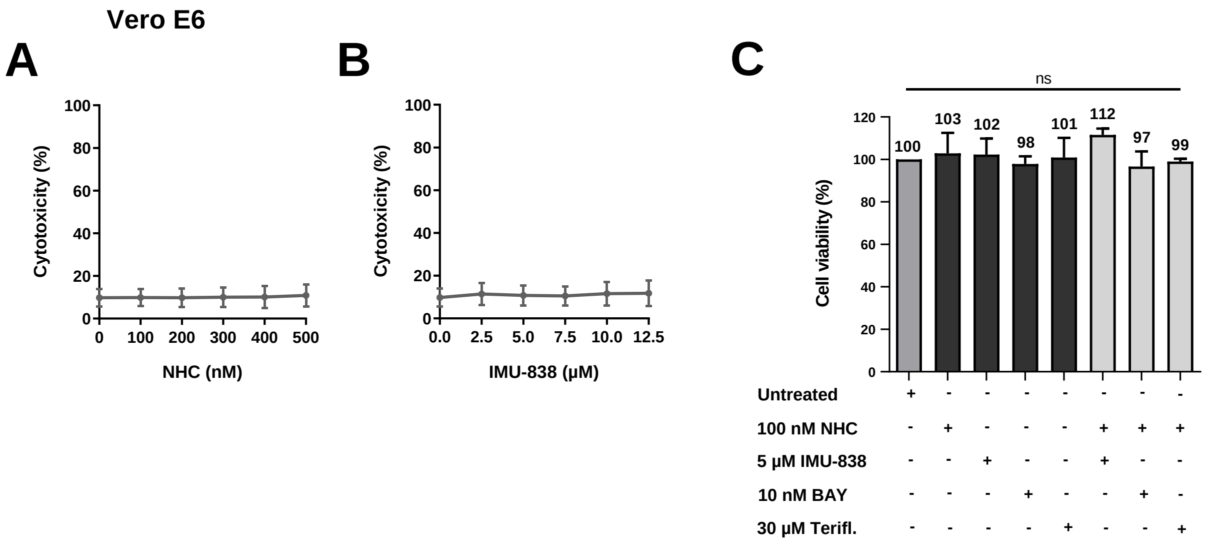

Figure S2, related to Figure 2

A

Vero E6

Simultaneous treatment and virus infection

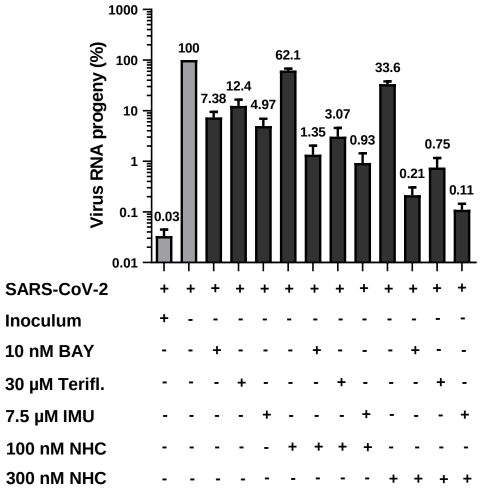

B

P values corresponding to Fig. S2A (Vero E6)

P value	Untreat.	10 nM BAY	30 µM Terifl.	7.5 µM IMU	100 nM NHC	100 nM +BAY	100 nM +Terifl.	100 nM +IMU	300 nM NHC	300 nM +BAY	300 nM +Terifl.	300 nM +IMU
Untreat.	n/a	<0.0001	<0.0001	<0.0001	0.0003	<0.0001	<0.0001	<0.0001	<0.0001	<0.0001	<0.0001	<0.0001
100 nM +BAY	<0.0001	0.0084	n/a	n/a	<0.0001	n/a	n/a	n/a	n/a	n/a	n/a	n/a
100 nM +Terifl.	<0.0001	n/a	0.0207	n/a	<0.0001	n/a	n/a	n/a	n/a	n/a	n/a	n/a
100 nM +IMU	<0.0001	n/a	n/a	0.0260	<0.0001	n/a	n/a	n/a	n/a	n/a	n/a	n/a
300 nM +BAY	<0.0001	0.0037	n/a	n/a	n/a	n/a	n/a	n/a	0.0002	n/a	n/a	n/a
300 nM +Terifl.	<0.0001	n/a	0.0081	n/a	n/a	n/a	n/a	n/a	0.0002	n/a	n/a	n/a
300 nM +IMU	<0.0001	n/a	n/a	0.0128	n/a	n/a	n/a	n/a	0.0002	n/a	n/a	n/a

C

P values corresponding to Fig. 2B (Vero E6)

P value	Untreat.	10 nM BAY	30 µM Terifl.	7.5 µM IMU	100 nM NHC	100 nM +BAY	100 nM +Terifl.	100 nM +IMU	300 nM NHC	300 nM +BAY	300 nM +Terifl.	300 nM +IMU
Untreat.	n/a	<0.0001	<0.0001	<0.0001	0.0080	<0.0001	<0.0001	<0.0001	<0.0001	<0.0001	<0.0001	<0.0001
100 nM +BAY	<0.0001	0.0066	n/a	n/a	0.0049	n/a	n/a	n/a	n/a	n/a	n/a	n/a
100 nM +Terifl.	<0.0001	n/a	0.0104	n/a	0.0045	n/a	n/a	n/a	n/a	n/a	n/a	n/a
100 nM +IMU	<0.0001	n/a	n/a	0.0260	0.0044	n/a	n/a	n/a	n/a	n/a	n/a	n/a
300 nM +BAY	<0.0001	0.0050	n/a	n/a	n/a	n/a	n/a	n/a	0.0019	n/a	n/a	n/a
300 nM +Terifl.	<0.0001	n/a	0.0077	n/a	n/a	n/a	n/a	n/a	0.0018	n/a	n/a	n/a
300 nM +IMU	<0.0001	n/a	n/a	0.0124	n/a	n/a	n/a	n/a	0.0018	n/a	n/a	n/a

Figure S3, related to Figure 4

Vero E6

A

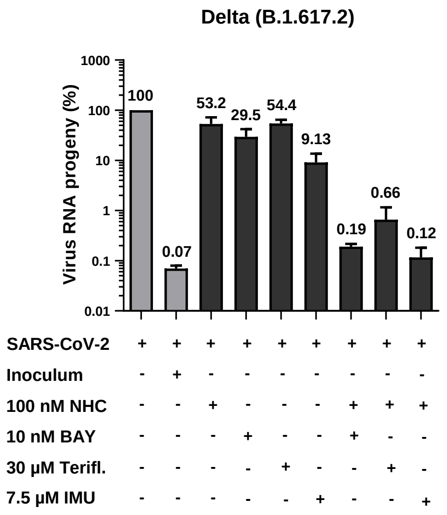

B **P values corresponding to Fig. S3A (Vero E6)**

P value	Untreated	100 nM NHC	10 nM BAY	30 µM Terifl.	7.5 µM IMU	NHC + BAY	NHC + Terifl.	NHC + IMU
Untreated	n/a	0.0125	0.0006	0.0014	<0.0001	<0.0001	<0.0001	<0.0001
NHC + BAY	<0.0001	0.0081	0.0157	n/a	n/a	n/a	n/a	n/a
NHC + Terifl.	<0.0001	0.0083	n/a	0.0008	n/a	n/a	n/a	n/a
NHC + IMU	<0.0001	0.0080	n/a	n/a	0.0248	n/a	n/a	n/a

Figure S4, related to Figure 5

A P values corresponding to Fig. 5A (Vero E6)

P value	Untreated	2 µM Uridine	5 µM Uridine	10 µM Uridine	100 nM NHC	7.5 µM IMU	NHC + IMU	Combi + 2 µM U.	Combi + 5 µM U.	Combi + 10 µM U.
Untreated	n/a	0.4600	0.2398	0.1390	0.0284	0.0013	<0.0001	<0.0001	0.0085	0.0185
NHC + IMU	<0.0001	0.0030	0.0039	0.0057	0.0160	0.0305	n/a	0.0727	0.0087	0.0124

P value	Untreated	2 µM Uridine	5 µM Uridine	10 µM Uridine	100 nM NHC	10 nM BAY	NHC + BAY	Combi + 2 µM U.	Combi + 5 µM U.	Combi + 10 µM U.
Untreated	n/a	0.1738	0.3976	0.3344	0.0743	0.3363	<0.0001	<0.0001	<0.0001	0.0483
NHC + BAY	<0.0001	0.0004	0.0043	0.0314	0.0122	0.0984	n/a	>0.9999	<0.0001	0.0400

P value	Untreated	2 µM Uridine	5 µM Uridine	10 µM Uridine	100 nM NHC	30 µM Terifl.	NHC + Terifl.	Combi + 2 µM U.	Combi + 5 µM U.	Combi + 10 µM U.
Untreated	n/a	0.3186	0.9366	0.8216	0.0004	0.0362	<0.0001	<0.0001	0.0116	0.0067
NHC + Terifl.	<0.0001	0.0069	0.0222	0.0450	0.0005	0.0001	n/a	0.0578	0.0280	0.0028

B P values corresponding to Fig. 5B (Vero E6)

P value	Untreated	2 µM Cytidine	5 µM Cytidine	10 µM Cytidine	100 nM NHC	7.5 µM IMU	NHC + IMU	Combi + 2 µM C.	Combi + 5 µM C.	Combi + 10 µM C.
Untreated	n/a	0.5520	0.2823	0.2097	0.0284	0.0013	<0.0001	<0.0001	0.0194	0.2010
NHC + IMU	<0.0001	0.0019	0.0044	0.0050	0.0160	0.0305	n/a	0.1161	0.0055	0.0060

P value	Untreated	2 µM Cytidine	5 µM Cytidine	10 µM Cytidine	100 nM NHC	10 nM BAY	NHC + BAY	Combi + 2 µM C.	Combi + 5 µM C.	Combi + 10 µM C.
Untreated	n/a	0.8512	0.1392	0.4292	0.0743	0.3363	<0.0001	<0.0001	0.0003	0.4395
NHC + BAY	<0.0001	0.0081	0.0054	0.0151	0.0122	0.0984	n/a	0.6433	0.0037	0.0269

P value	Untreated	2 µM Cytidine	5 µM Cytidine	10 µM Cytidine	100 nM NHC	30 µM Terifl.	NHC + Terifl.	Combi + 2 µM C.	Combi + 5 µM C.	Combi + 10 µM C.
Untreated	n/a	0.3045	0.0008	0.0096	0.0004	0.0362	<0.0001	<0.0001	0.0351	0.3677
NHC + Terifl.	<0.0001	<0.0001	<0.0001	0.0003	0.0005	0.0001	n/a	0.1627	0.0058	0.0034

Figure S5, related to Figure 7

A

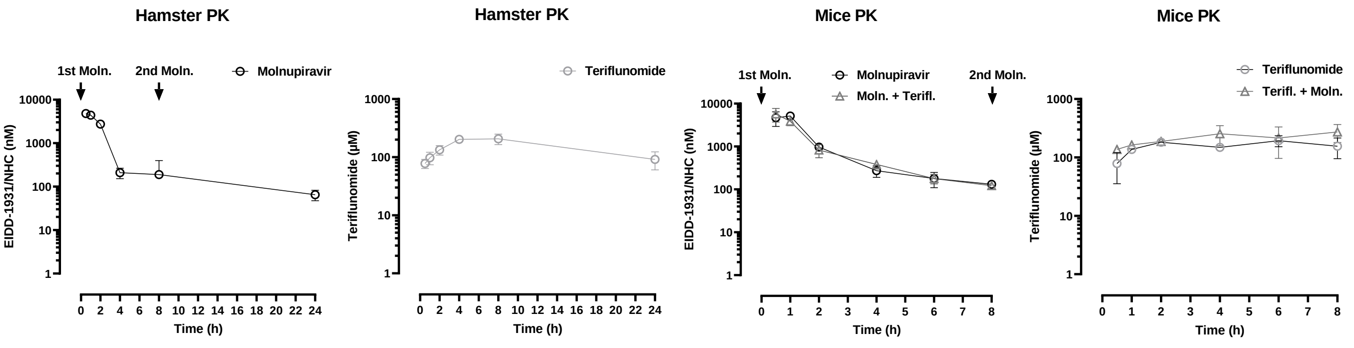

B

Syrian hamster model

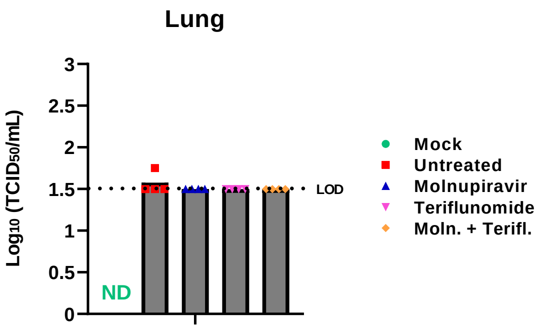

C

One-way ANOVA and Multiple Comparisons corresponding to Fig. 7C

One-way ANOVA

P value	3 dpi	4 dpi	5 dpi
Between Groups	0.000	0.000	0.000

Multiple Comparisons (Tukey post hoc test)

	3 dpi					4 dpi					5 dpi				
P value	Mock	Untr.	Moln.	Terif.	Moln. + Terif.	Mock	Untr.	Moln.	Terif.	Moln. + Terif.	Mock	Untr.	Moln.	Terif.	Moln. + Terif.
Mock	n/a	0.000	0.000	0.000	0.001	n/a	0.000	0.000	0.000	0.000	n/a	0.000	0.000	0.000	0.000
Untreated	0.000	n/a	0.077	1.000	0.017	0.000	n/a	0.180	1.000	0.017	0.000	n/a	0.027	0.905	0.002
Moln.	0.000	0.077	n/a	0.072	0.923	0.000	0.180	n/a	0.131	0.683	0.000	0.027	n/a	0.126	0.557
Terif.	0.000	1.000	0.072	n/a	0.016	0.000	1.000	0.131	n/a	0.012	0.000	0.905	0.126	n/a	0.008
Moln. + Terif.	0.001	0.017	0.923	0.016	n/a	0.000	0.017	0.683	0.012	n/a	0.000	0.002	0.557	0.008	n/a

Figure S6, related to Figure 7

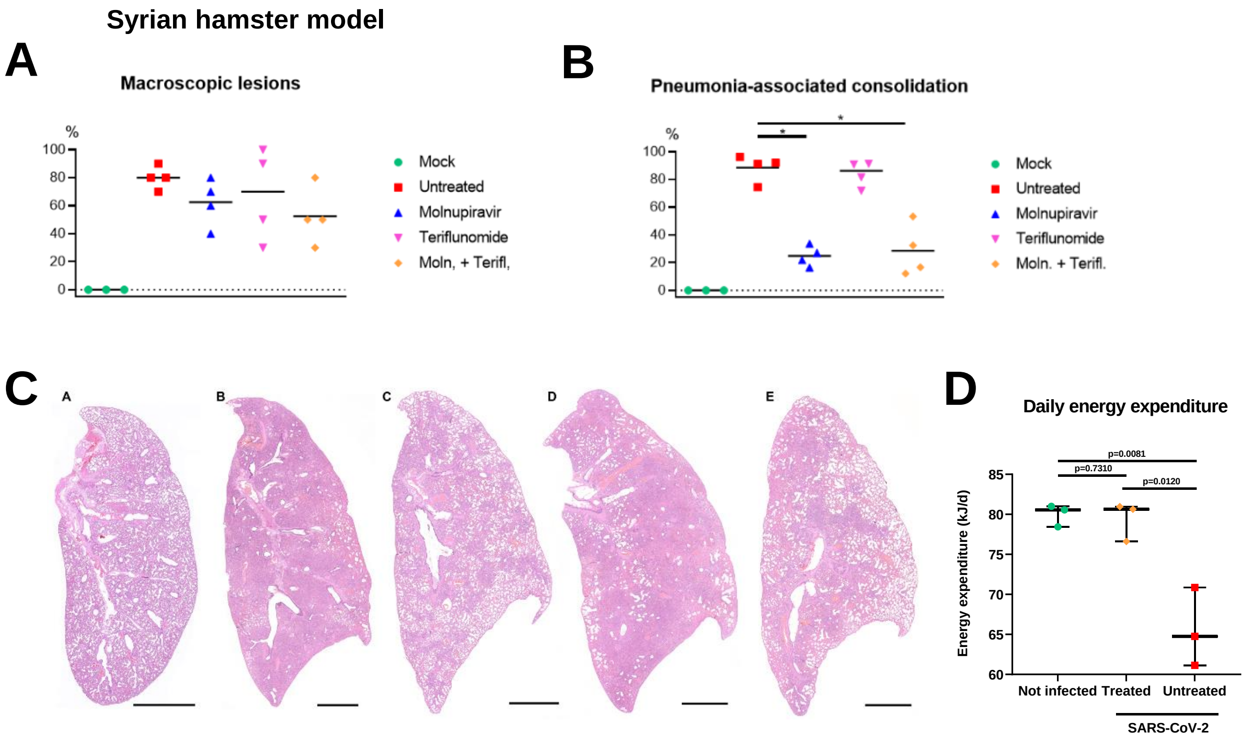

Figure S7, related to Figure 8

K18-hACE-2 mouse model

A

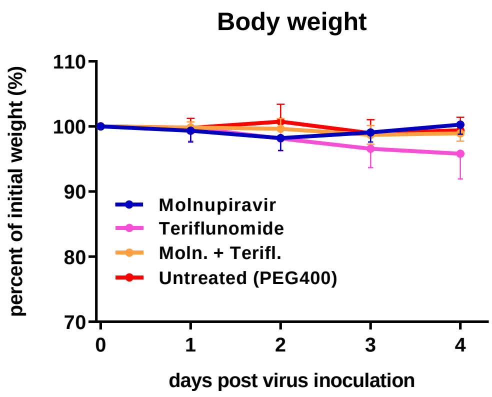

B P values corresponding to Fig. 8B

P value	Untreated	100 mg/kg/day Molnupiravir	10 mg/kg/day Terifl.	Moln. + Terifl.
Untreated	n/a	0.1009	0.3823	0.0202
Moln. + Terifl.	0.0202	0.1790	0.0204	n/a

C P values corresponding to Fig. 8C

P value	Untreated	100 mg/kg/day Molnupiravir	10 mg/kg/day Terifl.	Moln. + Terifl.
Untreated	n/a	0.6761	0.5068	0.0393
Moln. + Terifl.	0.0393	0.0631	0.1838	n/a

D

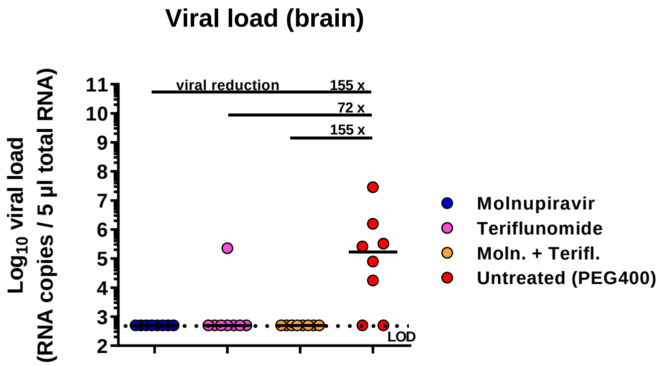

Supplement: Document S1. Figures S1–S7 [file mmc1.pdf]
